# Diet and Lipidomics Mediated Regulation of Mesenchymal Stem Cell Function: Diet, Omics and Stem Cell Connection

**DOI:** 10.3390/biom16081216

**Published:** 2026-08-20

**Authors:** Büşra Başar Gökcen, Büşra Atabilen Pınar, Menşure Nur Çelik, Zeynep Büşra Aksoy, Bence Raposa, Duygu Ağagündüz

**Affiliations:** 1 Department of Nutrition and Dietetics, Fethiye Faculty of Health Sciences, Muğla Sıtkı Koçman University, 48300 Mugla, Türkiye; busrabasar@mu.edu.tr; 2 Department of Nutrition and Dietetics, Faculty of Health Sciences, Karamanoğlu Mehmetbey University, 70200 Karaman, Türkiye; busraatabilen@kmu.edu.tr; 3 Department of Nutrition and Dietetics, Faculty of Health Sciences, Ondokuz Mayıs University, 55270 Samsun, Türkiye; mensurenur.celik@omu.edu.tr; 4 Stem Cell Institute, Ankara University, 06520 Ankara, Türkiye; zeynepbusraa@gmail.com; 5 Institute of Basics of Health Sciences, Midwifery and Health Visiting, Faculty of Health Sciences, University of Pécs, 7621 Pécs, Hungary; 6 Department of Nutrition and Dietetics, Faculty of Health Sciences, Gazi University, 06490 Ankara, Türkiye

**Keywords:** mesenchymal stem cells, lipid metabolism, lipidomics, cell differentiation, diet, obesity

## Abstract

Mesenchymal stem/stromal cells (MSCs) are promising candidates in regenerative medicine, but their effectiveness is significantly influenced by the surrounding metabolic and nutritional conditions. Increasing evidence suggests that lipids act not only as energy sources but also as regulators of MSC fate. This review explores how lipid metabolism influences the balance among stemness, immunomodulation, and differentiation into adipogenic or osteogenic lineages. It does so through mechanisms such as fatty acid uptake, β-oxidation, de novo lipogenesis, and membrane remodeling, all orchestrated by CD36, carnitine palmitoyltransferase 1A, PPARγ, AMP-activated protein kinase, and the PI3K/AKT/mTOR pathway. We then examine how diet reshapes the MSC lipidome: obesity and high-fat diets promote adipogenesis and senescence, while omega-3 fatty acids, caloric restriction, micronutrients, and a balanced microbiota help preserve regenerative capacity. Lastly, we discuss how combining lipidomics with multi-omics could uncover lipid-metabolic signatures and regulatory nodes that connect diet to MSC function. Overall, the diet–lipid–MSC axis emerges as a modifiable determinant of MSC function.

## 1. Introduction

MSCs are a heterogeneous, multipotent, non-hematopoietic progenitor cells of mesodermal origin with self-renewal, multilineage differentiation, tissue-regenerative, and immunomodulatory properties [1,2,3,4,5]. These cells can differentiate into osteoblasts, chondrocytes, and adipocytes, and are positive for CD73, CD90, and CD105 cell phenotypes, while they are negative for major histocompatibility complex class II (MHCII), CD11b, CD14, CD31, CD34, and CD45 [6,7]. Owing to their broad tissue distribution and accessibility, MSCs can be isolated from several sources, including bone marrow, adipose tissue, umbilical cord, dental pulp, placenta, the cervix, amniotic fluid, breast milk and synovial membrane [8]. Bone marrow-derived MSCs (BM-MSCs) are the most used stem cells in regenerative medicine and tissue engineering. They are non-hematopoietic stem cells found in bone marrow that possess multipotent differentiation potential [9]. The therapeutic use of BMSCs also has some limitations, such as cell rejection, poor immune response, toxicity, tumor formation, potential contamination with viruses, and problems related to cell transport and storage prior to transplantation [10,11]. In contrast, umbilical cord-derived MSCs (UC-MSCs) have a faster regeneration rate and are a better source than commonly used embryonic stem cells [12,13]. Adipose tissue-derived MSCs (AT-MSCs) have a wide range of clinical applications due to their easy availability from the stromal vascular fraction of adipose tissue and their anti-inflammatory and proangiogenic effects [14,15].

MSCs are a valuable therapeutic tool in the treatment of inflammatory, degenerative, and post-traumatic conditions due to their versatility, immunomodulatory properties, and paracrine activities. However, their therapeutic potential largely depends on the local microenvironment, which includes biochemical, metabolic, and nutritional factors that can modulate their biological functions [16]. With these characteristics, MSCs are demonstrating their potential in the treatment of diseases [17,18]. In some diseases (like non-alcoholic fatty liver disease and type II diabetic diseases), MSCs have been shown to prevent disease progression by regulating lipid metabolism [19,20]. These observations have increased interest in understanding how cellular lipid metabolism and lipid composition contribute to MSC function and therapeutic potential.

Lipids are essential structural and signaling molecules that contribute to membrane organization, energy storage, and regulation of cellular processes [21]. Glycerophospholipids, sphingolipids, and cholesterol constitute major components of cellular membranes, whereas fatty acids are important substrates for energy metabolism and lipid storage [22,23]. Furthermore, lipids act as signaling molecules through specific lipid–protein interactions. Signaling lipids, functioning as extracellular ligands or intracellular secondary messengers, are involved in regulating various biological processes such as cell proliferation, cell death, cell migration, gene expression, or inflammation [24]. The growing awareness of the role of lipids in metabolic diseases, technological advances in mass spectrometry and computational methods, and global efforts such as the LIPID MAPS (Metabolites and Pathways Strategy) consortium have propelled lipid biology to the forefront of metabolic research [24,25,26].

Lipids have also been shown to play important roles in the differentiation of stem cells [27]. Lipidome, a subset of metabolome, describes all the lipids found in a cell, tissue, or organism [28]. Lipidome, the total lipid content in a cell, is a dynamic phenomenon that can be actively remodeled under different physiological conditions, diets, and stimuli [29,30]. Any change in the cellular lipidome regulates signaling associated with cell function [26,31]. Lipidomic approaches comprehensively analyze the composition, dynamic changes, and interactions of lipids in organisms, revealing their roles in disease formation, development, and treatment [32,33]. Many studies have analyzed the lipidome of MSCs [31,34,35]. It is emphasized that small changes in membrane glycerophospholipids can have significant consequences for signaling transmitted via lipid derivatives; therefore, determining the changes caused by in vitro culture conditions is important, especially if stem cells are to be used for therapeutic purposes [26].

This review will first address the identification of MSC-specific lipidomic profiles and how these profiles enable dynamic remodeling. We will also highlight the role of genetic and nutritional-based modulation of lipid metabolism pathways, such as fatty acid uptake, de novo lipogenesis, and fatty acid oxidation (FAO), in regulating MSC behavior. Furthermore, we will examine the role of lipid-mediated signaling networks, such as eicosanoids, omega-3 polyunsaturated fatty acids, and lipid-sensitive nuclear receptors, in the regulation of MSCs. We will also investigate how lipids influence MSC characteristics through mechanisms such as metabolic reprogramming, epigenetic regulation, membrane dynamics, and asymmetric cell division. Finally, we will summarize the effects of nutritional changes, such as high-fat diets, calorie restriction, and microbiota-mediated lipid remodeling, on MSC function and tissue homeostasis. Through the integration of lipidomic data with nutritional and systems biology approaches, this review aims to provide a holistic view of the diet–lipid–MSC axis and to reveal its potential applications in regenerative medicine and metabolic health.

## 2. Mesenchymal Stem Cells: Functional Properties and Metabolic—Nutritional Regulation

### 2.1. Biological Characteristics and Functions of MSCs

MSCs are multipotent stem cells derived from diverse stromal and regenerative cell populations obtained from various tissues [36]. These cells were first identified in 1970 by Friedenstein et al. in a study investigating the development of fibroblast colonies in monolayer cultures of bone marrow and spleen cells derived from adult guinea pigs. The study demonstrated that fibroblast colony-forming cells (CFU-F or CFC-F) located within the bone marrow stroma function as progenitor cells with a high proliferative capacity [37]. Friedenstein subsequently proposed that osteogenic cells in the bone marrow originate from a precursor population referred to as mechanocytes, which includes fibroblasts, bone, cartilage, and reticular cells; he also introduced the term CFC-F to describe adherent cells capable of expansion in culture [38]. These cells were later investigated in greater detail by Caplan in 1991 and were subsequently termed “mesenchymal stem cells” [39].

According to the standards proposed by the Mesenchymal and Tissue Stem Cell Committee of the International Society for Cellular Therapy (ISCT), the identification of human mesenchymal stromal cells (MSCs) is based on three principal criteria. These criteria include: (1) adherence to plastic under standard culture conditions, (2) the expression of specific surface antigens—namely CD73, CD90, and CD105—while lacking the expression of CD11b, CD14, CD34, CD45, CD19 or CD79α, and human leukocyte anti-gen-DR (HLA-DR), and (3) multipotent differentiation potential [40]. Under in vitro conditions, these cells possess the capacity to differentiate into osteoblasts, adipocytes, and chondroblasts [6].

Nevertheless, another position paper published by the same committee of ISCT addressed the issue of terminological ambiguity. The paper emphasized that the terms “mesenchymal stem cell” and “mesenchymal stromal cell” should not be used interchangeably. Accordingly, it was proposed that the currently defined plastic-adherent cell populations should be referred to as “multipotent mesenchymal stromal cells.” In contrast, the term “mesenchymal stem cell” should be reserved exclusively for cell subpopulations in which stem cell activity—namely self-renewal and multilineage differentiation capacity in vivo—has been unequivocally demonstrated [41]. In this context, mesenchymal stem cells refer to a population exhibiting progenitor cell functions with the capacity for self-renewal and differentiation, whereas mesenchymal stromal cells represent a heterogeneous cell population characterized by prominent secretory, immunomodulatory, and homing properties [42,43]. In line with this distinction, it should be emphasized that the majority of studies discussed in this review were performed on plastic-adherent, culture-expanded populations that meet the minimal ISCT identity criteria but for which bona fide stem-cell activity, self-renewal and multilineage differentiation capacities in vivo, has not been formally demonstrated; such populations are therefore more accurately termed multipotent mesenchymal stromal cells. For consistency, the abbreviation “MSC” is used throughout this review to encompass both bona fide mesenchymal stem cells and the broader mesenchymal stromal cell compartment, and the qualifier “stromal” is retained wherever the cited evidence does not establish stemness. This terminological caution is widely acknowledged in the field, as reflected in the proposal to redesignate these cells as “medicinal signaling cells,” which better captures their predominantly paracrine, secretory, and trophic mode of action [44].

Their ease of in vitro expansion and relatively low ethical concerns, together with their multilineage potential [45,46], has attracted considerable interest in the development of MSC-based therapeutic strategies for the treatment of various human diseases [47,48]. The therapeutic effects of MSCs are generally categorized into six principal mechanisms: (1) immunomodulation, (2) inhibition of apoptosis, (3) stimulation of angiogenesis, (4) promotion of the proliferation and differentiation of local stem and progenitor cells, (5) inhibition of scar formation, and (6) regulation of cell migration (chemotaxis) toward the site of injury [49].

MSCs exhibit immunomodulatory properties, including both immune suppression and immune activation, together with low immunogenicity [50]. Indeed, MSCs are defined as immunosuppressive stromal cells present in numerous tissues and organs [51]. The immunomodulatory effects of MSCs are primarily mediated through direct cell–cell interactions with immune cells—including T cells, B cells, natural killer (NK) cells, macrophages, monocytes, dendritic cells, and neutrophils—and/or through paracrine mechanisms involving cytokines, chemokines, growth factors, and other bioactive molecules. Within the immune system, MSCs generally exert immunosuppressive effects that prevent excessive immune responses and regulate both innate and adaptive immune cells [36,45,52]. Zheng et al. demonstrated that co-culture of activated T cells with MSCs rapidly suppresses T-cell receptor (TCR) signaling and its downstream pathways through cell–cell contact mediated by the intercellular adhesion molecule-1 (ICAM-1)/CD43 interaction. In this process, MSCs were also shown to induce rapid Ca^2+^ signaling blockade in activated T cells, thereby reducing the gene expression of pro-inflammatory cytokines such as tumor necrosis factor-α (TNF-α) and interferon-γ (IFN-γ) [53]. Moreover, due to their low immunogenicity, MSCs rarely trigger immune responses and exhibit the capacity to migrate toward and home to sites of tissue injury, inflammation, and tumors [45,46,52]. MSCs produce various immunomodulatory molecules, including indoleamine 2,3-dioxygenase (IDO), transforming growth factor-beta (TGF-β), prostaglandin E2 (PGE2), and nitric oxide (NO), as well as immunosuppressive factors such as IL-10 (interleukin-10), CCL2 (chemokine ligand 2), PGE2 (prostaglandin E2), IL-1Ra (IL-1 receptor antagonist), M-CSF (macrophage colony-stimulating factor), TSG-6 (TNF-stimulated gene/protein 6), and HLA-G5 (human leukocyte antigen-G5) [46,54].

The microenvironment in which MSCs reside also significantly influences their immunomodulatory properties. MSCs possess an intrinsic ability to sense environmental alterations such as inflammation. Under such conditions, they can induce the release of biologically active agents and generate progenitor cells in response to these changes. High levels of inflammatory factors enhance the immunosuppressive effects of MSCs, whereas low levels of inflammatory stimuli may allow MSCs to exert immunostimulatory functions [48,50]. Indeed, He et al. demonstrated that lymph node-derived MSCs can exhibit immune-supportive properties. In contrast to BM-MSCs, lymph node-derived MSCs promote T-cell proliferation, differentiation, and activation through the production of high levels of Monocyte Chemoattractant Protein-1 (MCP-1), rather than suppressing T-cell activity [51]. MCP-1 exerts its effects by recruiting cells such as monocytes and/or macrophages to sites of inflammation and by enhancing the expression of other inflammatory factors and immune cells [55]. These findings suggest that MSCs derived from lymph nodes may play an immune-supportive rather than an immunosuppressive role, distinguishing them from other MSC subtypes [51]. In addition, MSCs secrete numerous factors that play critical roles in maintaining tissue homeostasis, supporting repair processes, and promoting regeneration. By releasing a wide variety of trophic factors with mitogenic, anti-apoptotic, neuroprotective/neurodegenerative, angiogenic, and other biological effects, MSCs accelerate the regeneration of damaged tissues. These factors include EGF (epidermal growth factor), VEGF (vascular endothelial growth factor), HGF (hepatocyte growth factor), PDGF (platelet-derived growth factor), Ang (angiopoietin), and IGF-1 (insulin-like growth factor-1) [36,54].

Although the therapeutic effects of MSCs were initially thought to depend primarily on their differentiation capacity and migratory ability, accumulating evidence indicates that these effects are mainly mediated through paracrine mechanisms, particularly via the secretome. The secretome refers to the complex repertoire of molecules and biological factors released by cells into the extracellular environment, including cytokines, chemokines, growth factors, and extracellular vesicles [56,57]. MSC-derived extracellular vesicles are generally spherical, well-defined structures surrounded by a phospholipid bilayer and characterized by a rich molecular cargo. Owing to their small size and lipid bilayer architecture, these vesicles are also capable of crossing biological barriers such as the blood–brain barrier [58]. These components can regulate cellular behavior through multiple mechanisms, including the enhancement of cell proliferation and differentiation, induction of anti-apoptotic, neuroprotective, and antibacterial protein gene expression, stimulation of chemotactic factor secretion, and promotion of immunomodulation [59]. Furthermore, these vesicles play a critical role in intercellular communication by enabling synergistic responses in recipient cells through cargos containing diverse proteins, lipids, and nucleic acids derived from the parent cells [60,61].

Depending on their tissue of origin, MSCs are classified into distinct subtypes and are generally derived from either adult or neonatal sources. The principal MSC types include BM-MSCs, AT-MSCs, UC-MSCs, dental pulp-derived MSCs (DP-MSCs), amniotic membrane-derived MSCs (AM-MSCs), synovial membrane-derived MSCs (SM-MSCs), and placenta-derived MSCs (PL-MSCs) [62,63,64]. From a functional perspective, AT-MSCs are more easily obtainable and provide high cell yields, whereas UC-MSCs exhibit high proliferative capacity and low immunogenicity. In contrast, BM-MSCs are the most extensively studied MSC subtype due to their superior differentiation potential and potent immunomodulatory effects [65]. BM-MSCs are of mesodermal origin and possess both self-renewal and multilineage differentiation capacities [66]. These MSCs are predominantly clustered within a central niche that constitutes approximately 90% of the total bone marrow area. This oxygen-rich microenvironment provides a highly vascularized niche and receives up to 10% of the total cardiac output, thereby contributing to the substantial energy demands of the human skeletal system [67].

### 2.2. Metabolic Networks Governing Differentiation

MSCs are classified into six principal lineage pathways according to their differentiation potential: osteogenic (bone), chondrogenic (cartilage), myogenic (muscle), adipogenic (adipose tissue), tendon/ligament fibroblastic, and stromal (connective tissue) lineages [49]. Among these, adipogenesis and osteogenesis are particularly significant, representing two closely interconnected mechanisms involved in determining cellular fate [68]. Indeed, the differentiation balance is tightly regulated between adipocytes and osteoblasts [69].

Adipogenesis proceeds in two steps: commitment of MSCs to the preadipocyte lineage, followed by maturation, during which cells accumulate lipid droplets and acquire the lipid-synthesizing, insulin-responsive phenotype of mature adipocytes [70,71,72,73]. When multipotent stem cells are directed toward the adipocyte lineage, they lose their ability to differentiate into other cell types and undergo extensive morphological and functional alterations mediated by numerous signaling pathways and transcription factors; this process is tightly regulated [74,75]. Upon induction, a first wave of the CCAAT/enhancer-binding proteins (C/EBP) C/EBPβ and C/EBPδ, acting downstream of cyclic adenosine monophosphate (cAMP) and the cAMP response element-binding protein (CREB), induces C/EBPα and peroxisome proliferator-activated receptor gamma (PPARγ), the master regulator of adipogenesis [73,76,77]. PPARγ is essential for adipogenesis and regulates the transcription of other related genes responsible for lipid uptake, transport, and storage [78]. Proliferation then ceases as the cells accumulate lipid droplets and up-regulate lipid-handling genes such as lipoprotein lipase (LPL), fatty acid synthase (FAS), adipose triglyceride lipase (ATGL), and perilipin, acquiring the lipid-storing, insulin-responsive phenotype of mature adipocytes [70,79].

The osteogenic differentiation process of MSCs consists of a series of stages involving the transition of mesenchymal progenitor cells into preosteoblasts, osteoblasts, and ultimately mature bone cells, accompanied by proliferation, lineage commitment, collagen secretion, and extracellular matrix mineralization, driven principally by runt-related transcription factor 2 (Runx2) and osterix (Sp7) [80,81,82,83]. Together with SRY-box transcription factor 9 (Sox9), these master factors determine the fate split between bone-forming osteoblasts and cartilage-forming chondrocytes. Sox9 initiates chondrogenic differentiation in skeletal progenitors, whereas Sp7 governs osteoblast precursor specification and Runx2 is required both for osteoblast lineage commitment and for the hypertrophic maturation of chondrocytes; accordingly, Runx2 and Sp7 direct MSCs toward the osteoblast lineage while Sox9 drives chondrogenesis [84,85].

The Wnt/β-catenin pathway is regarded as a critical checkpoint governing the balance between adipogenesis and osteogenesis and is one of the most extensively studied pathways in adipogenesis [85,86]; it is also considered a central regulator of MSC osteogenesis [87]. Its activation suppresses key adipogenic transcription factors such as C/EBPα and PPARγ, thereby inhibiting adipogenesis while promoting the differentiation of MSCs into osteoblasts [85,88]. Reciprocally, during osteogenic differentiation MSCs express Runx2 to drive osteogenic genes, whereas cells committing to the adipocyte fate express PPARγ [81].

Similarly, Hedgehog signaling inhibits adipocyte differentiation and can shift cell fate commitment from the adipogenic toward the osteogenic lineage [89]; it also exerts important regulatory effects on MSC osteogenesis, particularly at early stages, through Gli transcription factors [87]. In one study, adipose-tissue-derived exosomes activated Hedgehog signaling in AT-MSCs and suppressed adipogenic differentiation; this activation was necessary but not sufficient on its own, and the effect became particularly pronounced under high-fat conditions [90].

In contrast, bone morphogenetic proteins (BMPs) and their modulators were incorporated into the study of MSC lineage differentiation relatively later than the other pathways [91]. BMPs multifunctional cytokines of the TGF-β superfamily are recognized as principal regulators of osteoblast-specific differentiation and subsequent bone formation, and they also induce early chondrogenesis and drive the differentiation of MSCs into osteoblasts [92,93]. BMP and TGF-β act in opposition on adipogenesis: BMPs promote early (brown) adipocyte commitment, whereas TGF-β is inhibitory [94]. From a physiological standpoint, excessive commitment of BM-MSCs toward the adipocyte lineage reduces osteoblast numbers and relatively increases osteoclasts, ultimately disrupting bone homeostasis; this shift is promoted by lipid excess [66] and underlies the diet- and obesity-associated skeletal changes discussed in Section 6.

## 3. Lipid Metabolism and Metabolic Reprogramming in Mesenchymal Stem Cells

### 3.1. Fatty Acid Uptake and Intracellular Lipid Trafficking

Lipid metabolism, which plays a central role in stem cell physiopathology, is considered a critical determinant of MSC function and one of the key regulators of cellular proliferation [95,96]. The first regulatory step of nutrient homeostasis is the uptake of nutrients across the plasma membrane into the cell. This process is generally highly controlled and involves specific membrane receptors [97]. Although fatty acid uptake was long believed to occur through simple diffusion, it is now well established that specific proteins, such as cluster of differentiation 36 (CD36) and plasma membrane fatty acid-binding protein (FABPpm, also known as glutamic-oxaloacetic transaminase 2, GOT2), facilitate this process [98].

Fatty acids are internalized into MSCs through two principal pathways. First, triglyceride-rich lipoproteins, including very low-density lipoproteins (VLDL) and chylomicrons, bind to the cell surface through the VLDL receptor and heparan sulfate proteoglycans (syndecans), thereby initiating lipid uptake into the cell. Second, TAGs undergo hydrolysis via lipoprotein lipase (LPL), which is associated with syndecans on the MSC surface, and glycosylphosphatidylinositol-anchored high-density lipoprotein-binding protein 1 (GPIHBP1), located on the capillary endothelium, resulting in the release of free fatty acids. These fatty acids are subsequently transported into the cell through CD36 and G protein-coupled receptors (GPCRs) [67].

The principal functions of fatty acid translocase/CD36 include fatty acid uptake, cell adhesion, and activity as a class B scavenger receptor. Accordingly, CD36 acts as a specialized receptor for fatty acid uptake and plays an important role in the regulation of cellular metabolism [99]. Lin et al. (2026) [100], in a study investigating the therapeutic effects of BM-MSCs on pancreatic beta-cell dysfunction, examined the protective effects of BMSCs and their secreted extracellular vesicles on hypoxic beta cells. Their findings demonstrated that co-culture with BMSCs or extracellular vesicles significantly improved the viability and survival of hypoxic beta cells. This effect was associated with reduced lipid uptake, oxidative stress, and metabolic dysfunction mediated through the downregulation of CD36 gene expression. Another related study focused on atherosclerotic plaque formation and progression. Wang et al. (2015) [101] demonstrated that BM-MSCs promote the downregulation of scavenger receptors such as CD36 and class A scavenger receptor (SRA) in an ApoE-knockout mouse model. Since these receptors are responsible for oxidized LDL (oxLDL) uptake and play critical roles in initiating intracellular lipid accumulation, their downregulation reduces lipid uptake into cells. Consequently, foam cell formation in macrophages decreases and the development of atherosclerotic plaques is suppressed.

Additional studies have concentrated on cancer stem cells. For instance, Gyamfi et al. (2021) [99] demonstrated that genetic ablation of CD36 reduced adipocyte-induced epithelial–mesenchymal transition and stem cell-like properties. The study further revealed the existence of a positive feedback loop between CD36 and STAT3. Specifically, CD36 activates the STAT3 signaling pathway, whereas STAT3 binds to the CD36 promoter and regulates its gene expression. Increased CD36 expression was shown to trigger metabolic reprogramming toward enhanced fatty acid oxidation and to correlate with elevated FABP4 gene expression. Moreover, CD36 was found to interact directly with FABP4 to regulate intracellular fatty acid uptake, transport, and metabolism.

This interaction is associated with pronounced metabolic reprogramming during MSC differentiation into adipocytes, particularly at the stages of fatty acid uptake and intracellular transport. In this process, increased expression of proteins such as CD36 and FABP4 indicates enhanced fatty acid uptake and cytosolic trafficking [102]. In another study, CD36 was shown to promote adipogenesis under both in vivo and in vitro conditions. CD36 deficiency or downregulation impaired adipocyte differentiation and de novo adipose tissue formation independently of its role in macrophage infiltration [103]. Supporting these findings, Gao et al. demonstrated that CD36-positive human adipose-derived stromal cells exhibit pronounced adipogenic differentiation and lipid accumulation, while CD36 silencing attenuates lipid accumulation, indicating that CD36-mediated fatty acid uptake contributes to adipogenic commitment [104]. Another study investigated the effects of oxidized LDL (oxLDL) on MSC differentiation into osteoblasts. The findings demonstrated that CD36 mediates the suppressive effects of oxLDL on osteoblast differentiation of MSCs, and that this effect occurs through the Wnt signaling pathway, particularly via downregulation of β-catenin, a critical mediator of intracellular signal transduction [105].

Importantly, a clear distinction must be made between exogenous lipid uptake and endogenous lipid metabolism. While CD36 and related membrane transporters mediate the influx of exogenous lipids from the extracellular microenvironment to regulate cell fate decisions such as osteogenesis and adipogenesis [103,104,105], endogenous processes including de novo lipogenesis and fatty acid oxidation intrinsically control intracellular lipid synthesis, catabolism, and energetic switching. Recognizing these distinct roles is critical, as exogenous lipids primarily act as microenvironmental signals, whereas endogenous lipid dynamics dynamically dictate stem cell quiescence and self-renewal [26,106,107].

### 3.2. Fatty Acid Oxidation and Mitochondrial Bioenergetics

Lipid metabolism constitutes a critical regulatory mechanism governing whether a stem cell differentiates into a specific cell type or remains in an undifferentiated, self-renewing state [106]. In the context of stem cells, the regulation of fatty acid metabolism influences whether a stem cell maintains its undifferentiated pluripotent state or enters a lineage-specific differentiation program [108]. Importantly, the balance between lipid synthesis and degradation (lipogenesis and lipid oxidation) represents a fundamental determinant of stem cell fate [106]. In accordance with the general trends observed in stem cell populations, alterations in the balance between fatty acid oxidation (FAO) and de novo lipogenesis (DNL) influence the equilibrium between quiescent and proliferative cellular states [107]. Contrary to the long-standing assumption that stem cells are predominantly dependent on glycolysis, FAO is now recognized as a key factor in the maintenance of adult stem cell populations. Nevertheless, the beneficial effects of FAO may vary depending on the stem cell type and its behavioral characteristics, such as whether the cells are quiescent or rapidly proliferating [26].

In adult stem cells, FAO is tightly regulated by pathways such as AMP-activated protein kinase (AMPK) and peroxisome proliferator-activated receptor alpha (PPARα), which sense cellular energy levels and lipid availability. These pathways exert their effects primarily through the carnitine palmitoyl transferase (CPT) system, the principal regulatory mechanism responsible for transporting long-chain fatty acids into the mitochondrial matrix for subsequent oxidation [108,109]. The CPT enzyme system consists of two isoforms localized within mitochondria: CPT I and CPT II. CPT I, a transmembrane protein, is located on the outer mitochondrial membrane, whereas CPT II resides within the mitochondrial matrix [110]. Long-chain fatty acids are first converted into acyl-CoA by long-chain acyl-CoA synthetase, after which the CPT system transports acyl-CoA from the cytoplasm into the mitochondrial matrix. CPT I convert acyl-CoA into acylcarnitine, and carnitine-acylcarnitine translocase (CACT) mediates the exchange of acylcarnitine and carnitine across the outer and inner mitochondrial membranes. Finally, acylcarnitine is reconverted into acyl-CoA by CPT II, rendering it available for β-oxidation [111].

A recent study demonstrated that MSC administration exerts protective effects against renal ischemia/reperfusion injury by modulating lipid metabolism. Intravenous administration of rat BM-MSCs via the tail vein prior to renal ischemia/reperfusion injury in rats resulted in downregulation of fatty acid biosynthesis after 48 h of reperfusion. This metabolic shift toward lipid catabolism was associated with reduced levels of the lipid peroxidation products malondialdehyde and 4-hydroxy-2-nonenal in ischemic renal tissue [112]. Another investigation examined the effects of MSCs on reducing lipid accumulation in hepatocytes within a non-alcoholic steatohepatitis (NASH) model. In this study, BM-MSCs were transplanted via splenic injection into immunodeficient mice with established NASH, and the treatment significantly reduced hepatocellular lipid accumulation. This effect is thought to occur through direct interactions between MSCs and hepatocytes via structures known as tunneling nanotubes, which facilitate mitochondrial transfer. The transfer of healthy mitochondria enhances energy production in hepatocytes, promotes lipid oxidation, and thereby reduces intracellular lipid accumulation [113]. Similarly, Xue et al. demonstrated that human UC-MSCs significantly reduced lipid accumulation and triglyceride content in HepG2 cells, a human hepatocellular carcinoma cell line. This effect was primarily attributed to mitochondrial transfer from UC-MSCs. Tunneling nanotubes (TNTs) formed between MSCs and HepG2 cells were shown to play a critical role in the intercellular transfer of functional mitochondria. Mitochondrial transfer enhanced oxidative metabolism in recipient cells, thereby promoting more efficient lipid metabolism through β-oxidation. These findings further demonstrated that the regulatory effects of MSCs on lipid metabolism depend not only on paracrine factors but also on direct intercellular interactions mediated by organelle transfer [114]. Another study reported that MSC administration contributes to the metabolic reprogramming of lipid metabolism through activation of the mechanistic target of rapamycin (mTOR) pathway. In a rat model of liver failure induced by 90% hepatectomy, portal vein injection of rat BM-MSCs was shown to restore mitochondrial function, enhance fatty acid β-oxidation, and consequently reduce lipid accumulation in hepatocytes [115]. Similarly, in a rat model of thioacetamide-induced ovarian injury, intravenous administration of human PL-MSCs via the tail vein reduced intracellular lipid accumulation and reprogrammed lipid metabolism. This effect was particularly associated with increased expression of CPT1A, the rate-limiting enzyme responsible for mitochondrial fatty acid transport and β-oxidation [116].

### 3.3. De Novo Lipogenesis and Membrane Remodeling

De novo lipogenesis is a fundamental metabolic process that supports stem cell activity and differentiation. In particular, by meeting the increased demand for membrane biosynthesis during cellular proliferation, this process enables the rapid expansion of plasma and organelle membranes [26]. Membrane lipids, including fatty acid components, regulate numerous critical aspects of stem cell physiology, maintenance of stemness, and differentiation processes [34].

In a study revealing previously underappreciated effects of MSCs on lipid metabolism, Frodermann et al. demonstrated in a C57BL/6 male mouse model that intravenous administration of syngeneic murine BM-MSCs suppressed the de novo hepatic lipogenesis pathway. Specifically, significant reductions in the expression of stearoyl-CoA desaturase-1 (SCD1), and SREBP-2, which is associated with cholesterol synthesis, were observed in the intervention group [117]. SCD1 is an integral membrane enzyme localized to the endoplasmic reticulum that plays a critical role in regulating cellular lipid composition by converting saturated fatty acids into monounsaturated fatty acids (MUFAs). From the perspective of stem cell biology, this SCD1-mediated conversion represents an important mechanism influencing the metabolic state and differentiation capacity of MSCs. In this context, increased SCD1 activity may contribute to metabolic reprogramming in MSCs, thereby modulating cellular proliferation and differentiation potential. Indeed, enhancement of SCD1 expression and the resulting shift in lipid composition toward MUFA enrichment have been shown to create a microenvironment supportive of endothelial differentiation and to enhance the functional capacity of stem cells [118]. In contrast, Lee et al. demonstrated that the principal enzyme regulating the proliferation and migration of umbilical cord blood (UCB)-derived human MSCs (hMSCs) is not SCD1, but rather fatty acid synthase (FASN). This regulation was shown to be controlled through the hypoxia-inducible factor-1α (HIF-1α)/SREBP cleavage-activating protein (SCAP)/SREBP1 signaling pathways. The study further demonstrated that hypoxia-induced alterations in lipid metabolism play a crucial role in regulating cell fate and function [119]. Another study demonstrated that the pronounced M2-like polarization induced by AT-MSCs is associated with increased expression of lipid droplet biogenesis. Conditioned medium derived from murine adipose tissue-derived MSCs enhanced phosphorylation of AKT/mTOR pathway proteins in macrophages. Furthermore, macrophages exhibiting an M2-like phenotype—characterized by elevated arginase-1 expression and increased IL-10 levels—displayed increased PPARγ expression, enhanced lipid droplet biogenesis, and elevated PGE2 synthesis [120].

The clinical application of human mesenchymal stem/stromal cells (hMSCs) requires serial expansion of the cells, which represents another major area of investigation. In this context, one study examined the effects of cell division number on the glycerophospholipid (GPL) profile of BM-MSCs and demonstrated marked alterations in lipid composition during progressive culture passaging. Late-passage cells exhibited increased phosphatidylinositol/phosphatidylserine ratios and accumulation of PC and PE species containing 20:4n − 6, whereas species containing monounsaturated fatty acids were reduced. In addition, arachidonic acid (20:4n − 6) levels increased within the total fatty acid pool, largely at the expense of n − 3 polyunsaturated fatty acids (PUFAs), particularly DHA (22:6n − 3). These findings indicate that prolonged cell expansion adversely modulates membrane GPL composition, disrupts lipid signaling, and ultimately impairs cellular functions [121]. Moreover, a study investigating endometabolomic and exometabolomic dynamic adaptations demonstrated that lipid metabolism undergoes time-dependent reprogramming during osteogenic differentiation of human amniotic MSCs (hAMSCs). During the early phase (days 0–1), increased levels of 1-monoglycerides (1-MGs) were observed at the expense of mobilization of fatty acids from TAGs and GPLs, accompanied by reductions in PC and MUFA levels. Between days 1 and 7, 1-MGs were consumed, which was proposed to support triglyceride accumulation and consequently contribute to energy storage through lipid droplets. Simultaneously, a marked increase in unsaturated fatty acids, particularly MUFAs and ω-6 PUFAs, was observed, suggesting a protective adaptive mechanism aimed at reducing the potential cytotoxic effects of saturated fatty acids. Additionally, increased levels of phospholipid species—including PCs, PEs, plasmalogens, and sphingomyelins—were detected during days 1–7, changes that were associated with enhanced phospholipid biosynthesis supporting membrane remodeling [122].

Lipids also play critical roles in multiple stages that determine the fate and biological activity of MSC-derived exosomes. First, lipids participate directly in exosome biogenesis, while exosomes themselves carry numerous bioactive lipids as well as enzymes associated with lipid metabolism. Exosomes contain a broad spectrum of free fatty acids either synthesized within their own structures or incorporated during biogenesis [49]. Furthermore, the membranes of MSC-derived exosomes are highly enriched in lipid components, enabling them to fuse efficiently with target cells and transfer their molecular cargo into recipient cells [50].

### 3.4. Lipid-Driven Metabolic Switching

The metabolic plasticity of MSCs represents a fundamental mechanism underlying their therapeutic potential, enabling adaptation to changing microenvironmental conditions while maintaining their functional properties [123]. In this context, pH and oxygen tension, which constitute key components of the cellular microenvironment, emerge as critical determinants of stem cell fate and cellular viability [124]. The average partial oxygen pressure within natural human stem cell niches is generally considered to range between 3% and 6% [125]. Due to long-term evolutionary adaptation to this hypoxic niche, stem cells predominantly rely on anaerobic energy metabolism [126]. Under hypoxic niche conditions, hypoxia-inducible factors (HIFs) reduce the expression of mitochondrial enzymes while increasing the expression of glycolytic enzymes and glucose transporters [127]. Consequently, cells preferentially utilize anaerobic glycolysis rather than oxidative phosphorylation for energy production, resulting in suppression of mitochondrial activity [125]. Nevertheless, a study conducted on UC-MSCs demonstrated that these cells develop a metabolic adaptation aimed at directing excess lipids toward β-oxidation in response to increased lipid load. In this study, which highlighted the influence of the maternal metabolic environment on MSC lipid metabolism, FAO, and metabolic flexibility, MSCs were classified into three distinct metabolic phenotypes according to their triglyceride storage capacities. The findings revealed that the phenotype with high storage capacity was associated with elevated levels of AMP-activated protein kinase (AMPK) activity and FAO, both of which are closely linked to cellular energy metabolism [128].

Within their native microenvironment, MSCs exist in a quiescent state characterized by low proliferative activity and high multipotency, during which stem cells preserve “young” mitochondria through active autophagy and mitophagy [129]. Compared with cells committed to differentiation, self-renewing stem cells possess fewer mitochondria, which are fragmented and exhibit simpler cristae structures. This mitochondrial morphology is consistent with glycolytic dependency, resembling the metabolic adaptation observed in cancer cells [130]. Such adaptation may protect cells from oxidative damage caused by mitochondrial reactive oxygen species (ROS), thereby preventing injury to genetic material and cellular components [131]. High glycolytic flux additionally exerts cytoprotective effects by increasing the production of antioxidant precursors through the pentose phosphate pathway [129]. Furthermore, pyruvate generated through glycolysis is preferentially converted into lactate due to high lactate dehydrogenase (LDH) activity, while the NAD^+^ generated during this process serves as a critical cofactor required for sustaining stem cell proliferation [130]. Overall, this metabolic shift preserves stem cell self-renewal capacity and multilineage differentiation potential while supplying cofactors and substrates required for biosynthetic reactions essential for cellular proliferation, such as nucleotide and serine biosynthesis [132].

The differentiation and proliferation processes of MSCs require extensive metabolic adaptations [133]. In the context of stem cells, regulation of fatty acid metabolism influences whether a stem cell maintains its undifferentiated pluripotent state or enters a lineage-specific differentiation program [108]. In undifferentiated stem cells, more than 97% of adenosine triphosphate (ATP) production is derived from glycolysis, whereas the combined contribution of glucose and fatty acid oxidation accounts for only approximately 3% [134]. However, osteogenic differentiation of MSCs has been shown to activate mitochondrial oxidative phosphorylation while maintaining glycolytic activity at comparable levels. While undifferentiated MSCs exhibit high HIF-1 expression, osteogenically induced (differentiated) MSCs display reduced HIF-1 gene expression, and this reduction has been shown to be necessary for activation of oxidative phosphorylation [135]. Another study demonstrated that mitochondrial dynamics play a critical role during MSC differentiation. During adipogenesis, mitochondrial biogenesis increases, whereas the early stages of adipogenic and osteogenic differentiation are associated with elevated expression of genes related to mitochondrial fusion. In contrast, chondrogenesis is characterized by increased expression of genes associated with mitochondrial fission [136]. Furthermore, MSC differentiation induces plasma membrane remodeling, leading to the establishment of distinct lipid compositions [137].

Beyond their metabolic preferences associated with proliferation and differentiation within their native niches, activated MSCs undergo extensive metabolic reprogramming to support specific functional activities by regulating energy production pathways. One such process is related to their immunomodulatory functions. The potent immunomodulatory capacity of MSCs is associated with high metabolic demands and is directly linked to a metabolic shift toward aerobic glycolysis [123]. Stimulation by pro-inflammatory factors such as tumor necrosis factor-alpha (TNF-α) and interferon-gamma (IFN-γ) has been shown to induce rapid glucose consumption and a metabolic shift toward glycolysis in MSCs. This metabolic adaptation increases the expression of indoleamine 2,3-dioxygenase (IDO) and TNF-stimulated gene-6 (TSG-6). Furthermore, the glycolysis-dependent anti-inflammatory properties acquired by MSCs in response to inflammatory cytokines were shown to be regulated through the phosphoinositide 3-kinase/protein kinase B (PI3K-AKT) signaling pathway [138].

Similarly, another study demonstrated that IFN-γ-induced immune polarization reshapes MSC metabolic pathways toward glycolysis. This study further showed that activation of the Akt (protein kinase B)-mTOR pathway, downstream of PI3K-AKT signaling, is required for this process. Exposure to IFN-γ was found to suppress the mitochondrial electron transport chain, while increased ROS levels functioned as signaling mediators in this metabolic reprogramming process [139]. The PI3K/AKT/mTOR signaling pathway is a central intracellular signaling cascade regulating cellular growth, metabolism, and proliferation. Activation of PI3K triggers AKT activation, which subsequently regulates numerous downstream targets controlling cellular processes, whereas mTOR complexes modulate cellular growth and metabolic responses according to nutrient availability [140]. Yao et al. identified the Janus kinase (JAK)-signal transducer and activator of transcription (STAT) pathway as a novel crosstalk mechanism linking glucose metabolism, mitochondrial oxidation, and immunomodulatory functions in IFN-γ-stimulated human UC-MSCs. IFN-γ stimulation was shown to suppress lactic acid glycolysis while enhancing mitochondrial aerobic oxidation, thereby dynamically regulating intracellular ATP levels. ATP production subsequently promoted IFN-γ-mediated activation of the JAK-STAT pathway, leading to increased expression of indoleamine 2,3-dioxygenase (IDO) and programmed death-ligand 1 (PD-L1) [141]. The JAK/STAT signaling pathway constitutes a central communication network regulating cellular functions and participates in numerous biological processes, including hematopoiesis, immune responses, tissue repair, inflammation, apoptosis, and adipogenesis [142].

MSC stimulation with TNF-α and IFN-γ has also been shown to induce marked remodeling of lipid composition. In this context, increases in PC species containing long-chain fatty acids—particularly PC (36:1) and PC (38:4)—were observed, whereas PC species containing short-chain fatty acids were reduced. Parallel to these changes, elevated stearic acid levels accompanied by decreased palmitic acid levels suggested enhanced activity of elongation of very long chain fatty acids protein 6 (ELOVL6), an enzyme involved in long-chain fatty acid elongation. Additionally, reduced PC (40:6) levels together with increased PC (18:0) levels suggested enhanced phospholipase A2 (PLA2) activity under inflammatory conditions, leading to the cleavage of PC (40:6) and release of PC (18:0) and the C22:6n − 3 fatty acid. The increased abundance of PC(18:0), which possesses known anti-inflammatory properties, suggests that this lipid species may play a significant role in MSC immunomodulatory mechanisms [31]. Another study demonstrated that hepatic differentiation of MSCs is accompanied by decreased levels of major polyunsaturated fatty acids (PUFAs), whereas maturation is associated with increased levels of saturated fatty acids (SFAs). These findings suggest that hepatocyte formation during stem cell differentiation is associated with a shift in fatty acid profiles [118].

**Figure 1:**
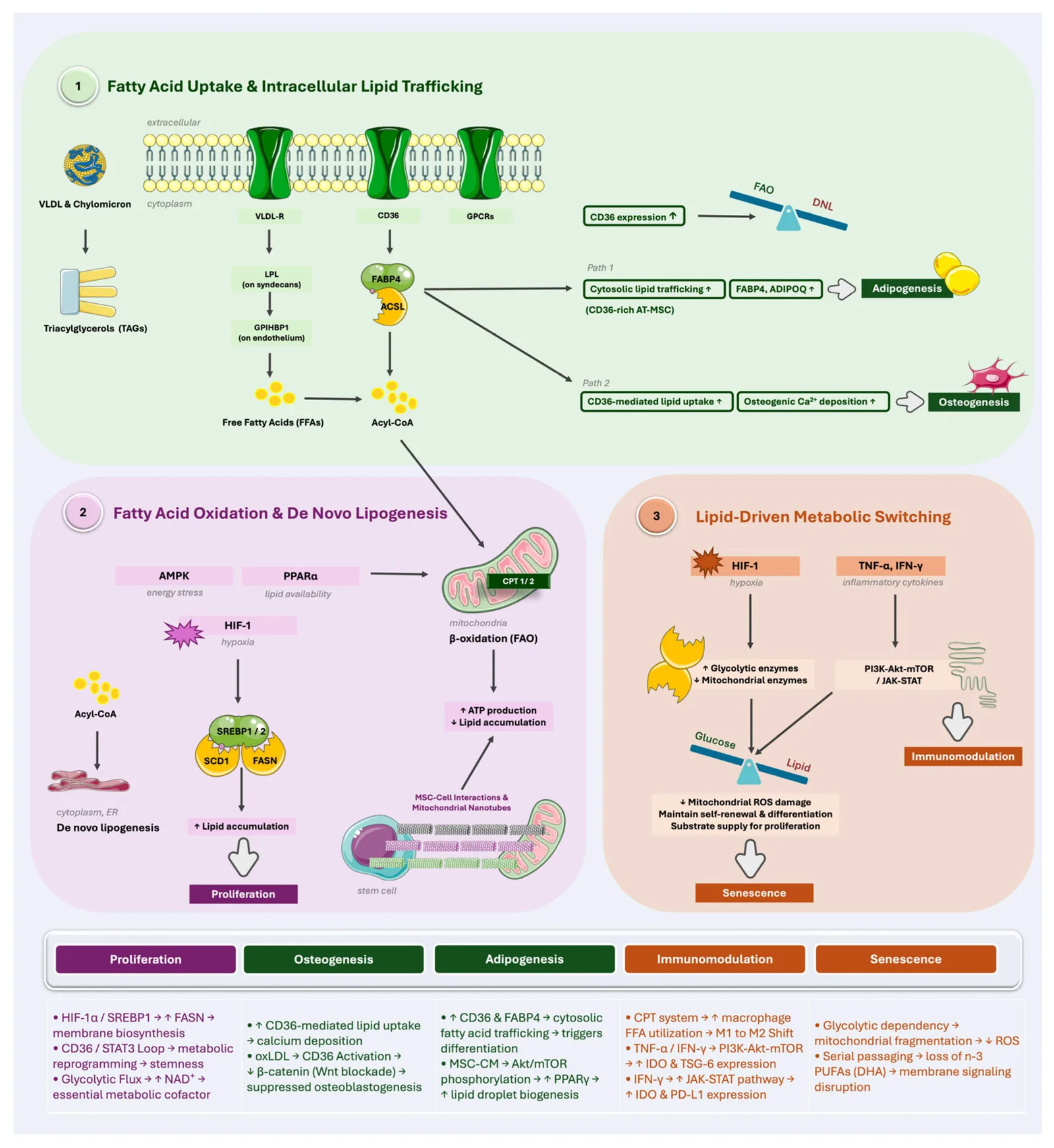
Lipid metabolism and metabolic reprogramming govern MSC function.ACSL, acyl-CoA synthetase (long-chain); ADIPOQ, adiponectin; AKT, protein kinase B; AMPK, AMP-activated protein kinase; AT-MSC, adipose tissue-derived MSC; ATP, adenosine triphosphate; CD36, cluster of differentiation 36 (fatty acid translocase); CPT I/II, carnitine palmitoyltransferase I/II; DHA, docosahexaenoic acid; DNL, de novo lipogenesis; ER, endoplasmic reticulum; FABP4, fatty acid-binding protein 4; FAO, fatty acid oxidation; FASN, fatty acid synthase; FFA, free fatty acid; GPCR, G protein-coupled receptor; GPIHBP1, glycosylphosphatidylinositol-anchored high-density lipoprotein-binding protein 1; HIF-1α, hypoxia-inducible factor-1α; IDO, indoleamine 2,3-dioxygenase; IFN-γ, interferon-γ; JAK-STAT, Janus kinase–signal transducer and activator of transcription; LPL, lipoprotein lipase; M1/M2, classically/alternatively activated macrophage phenotypes; MSC, mesenchymal stem/stromal cell; MSC-CM, MSC-conditioned medium; mTOR, mechanistic target of rapamycin; NAD^+^, nicotinamide adenine dinucleotide; n − 3, omega-3; oxLDL, oxidized low-density lipoprotein; PD-L1, programmed death-ligand 1; PI3K, phosphoinositide 3-kinase; PPARα/γ, peroxisome proliferator-activated receptor α/γ; PUFA, polyunsaturated fatty acid; ROS, reactive oxygen species; SCD1, stearoyl-CoA desaturase-1; SREBP1/2, sterol regulatory element-binding protein 1/2; STAT3, signal transducer and activator of transcription 3; TAG, triacylglycerol; TNF-α, tumor necrosis factor-α; TSG-6, TNF-stimulated gene-6; VLDL, very-low-density lipoprotein; VLDL-R, VLDL receptor; Wnt, wingless-related integration site (Wnt signaling); β-catenin, beta-catenin. Lipid metabolism and metabolic reprogramming govern mesenchymal stem cell (MSC) function. (1) Fatty acid uptake and intracellular trafficking: triglyceride-rich lipoproteins (VLDL, chylomicrons) are hydrolyzed by LPL/GPIHBP1 to release free fatty acids, which enter through CD36, FABPpm and GPCRs and are shuttled by FABP4/ACSL to acyl-CoA; CD36–FABP4 signaling drives adipogenesis (FABP4, ADIPOQ) and modulates osteogenesis. (2) Fatty acid β-oxidation and de novo lipogenesis: AMPK/PPARα and the CPT system route acyl-CoA into mitochondrial β-oxidation (↑ ATP, ↓ lipid accumulation), while HIF-1α/SREBP1–FASN/SCD1 support de novo lipogenesis and membrane remodeling; mitochondrial transfer via tunneling nanotubes reprograms recipient-cell metabolism. (3) Lipid-driven metabolic switching: hypoxia (HIF-1α) and inflammatory cytokines (TNF-α, IFN-γ) shift MSCs between glycolysis and oxidative phosphorylation via PI3K-Akt/mTOR and JAK-STAT, tuning immunomodulation and senescence. Solid black arrows indicate metabolic flux, sequential conversion, or activation; gray block arrows indicate the resulting MSC function. Upward (↑) and downward (↓) symbols denote increases and decreases in the indicated molecule or process, respectively. The lower panel links each lipid-metabolic pathway to core MSC functions. The figure were created using the sources in Section 3. Image(s) provided by Servier Medical Art (https://smart.servier.com), licensed under CC BY 4.0 (https://creativecommons.org/licenses/by/4.0/, accessed on 18 August 2026).

In conclusion, lipid metabolism in mesenchymal stem cells plays a central role in regulating cellular energy homeostasis, proliferation, differentiation, and immunomodulatory functions through multidimensional processes including fatty acid uptake, β-oxidation, de novo lipogenesis, and membrane remodeling. Metabolic regulators such as CD36, CPT1A, and PI3K/AKT/mTOR appear to be critically important for MSC adaptation to microenvironmental conditions and metabolic reprogramming. Collectively, these findings demonstrate that lipid metabolism functions not merely as a source of cellular energy, but also as a fundamental biological control mechanism determining stem cell fate, therapeutic efficacy, and regenerative capacity. These interconnected processes are summarized in Figure 1, which links each lipid-metabolic pathway—fatty acid uptake, β-oxidation, de novo lipogenesis, and lipid-driven metabolic switching—to the core MSC functions of proliferation, osteogenesis, adipogenesis, senescence, and immunomodulation, with respective experimental findings detailed in Table 1.

## 4. Lipid-Mediated Signaling Networks in Mesenchymal Stem Cells Regulation

Lipids are fundamental to both cellular physiology and pathological processes. They constitute the primary structural elements of cell membranes. Beyond this basic role, lipids function as energy reservoirs, precursors of signaling molecules, and scaffolds that facilitate protein localization and interaction [143]. Lipid metabolism also plays a crucial role in the physiology and pathology of mesenchymal stem cells (MSCs) [95]. Therefore, elucidating the influence of lipid metabolism on MSC behavior provides valuable insights for developing new strategies to regulate stem cell function both in vitro and in vivo [26]. This section has described lipid-mediated signaling networks and their role in the regulation of MSCs.

### 4.1. Eicosanoids and Prostaglandin Pathways

Eicosanoids are oxygenated metabolites derived from 20-carbon polyunsaturated fatty acids, with arachidonic acid serving as the principal precursor. They are generated through three main enzymatic pathways: cyclooxygenase, lipoxygenase, and cytochrome P450. The cyclooxygenase pathway produces prostaglandins and thromboxanes (altogether known as prostanoids), the lipoxygenase pathway yields leukotrienes and lipoxins, and the cytochrome P450 pathway generates a range of epoxy, hydroxy, and dihydroxy derivatives [144]. Arachidonic acid–derived eicosanoids are well recognized for their pleiotropic roles in inflammation. They regulate processes such as leukocyte chemotaxis and activation, induction of fever and pain, modulation of vascular permeability and tone, and platelet aggregation. Prostanoids also sustain chronic inflammation by amplifying cytokine signaling and promoting pro-inflammatory immune responses [145].

Within this inflammatory context, MSCs are known to actively produce lipid mediators that modulate immune responses. Prostaglandin E2 (PGE2) is currently considered one of the most important immunomodulatory factors secreted by MSCs. However, PGE2 secretion is influenced by the microenvironment and is increased under inflammatory conditions [146]. It has been shown that low levels of PGE2 exert pro-inflammatory effects, whereas higher levels display anti-inflammatory activity. PGE2 can exert pro-inflammatory effects by promoting dendritic cell maturation and enhancing T-cell proliferation. By interacting with PGE2 receptor 2 (EP2) and EP4 receptors on immune cells, PGE2 promotes the production of anti-inflammatory cytokines and suppresses pro-inflammatory responses, leading to the upregulation of cyclic AMP and subsequent activation of the protein kinase A and phosphatidylinositol-3 kinase pathways [147]. Furthermore, PGE2 can promote M2 macrophage polarization, inhibits natural killer cell activity, suppresses dendritic cell maturation, and regulates T and B cell functions toward a more anti-inflammatory profile [146]. In a related study, macrophages and dendritic cells were co-cultured with adipose derived-MSCs under non-contact conditions. The findings demonstrated that MSC co-culture enhanced the expression of phagocytic receptors, modulated chemokine receptor profiles, and reduced the secretion of pro-inflammatory cytokines in both macrophages and dendritic cells. These effects were primarily mediated by PGE2 secretion from MSCs [148]. Another study showed that intravenous administration of adipose-derived MSCs via the tail vein significantly improved myocardial fibrosis and cardiac dysfunction in rats with induced diabetic cardiomyopathy. However, in rats that received PGE2-deficient MSC transfer, the capacity for improvement in cardiac fibrosis and dysfunction was reduced [149]. Moreover, PGE2 contributes to improving the therapeutic effectiveness of MSC-based treatments. In a study evaluating the immunomodulatory effects of PGE2, human PL-MSCs preconditioned with PGE2 were administered intravenously to rats with experimentally induced acute lung injury. The results demonstrated that PGE2-treated MSCs effectively alleviated lung injury and increased the production of anti-inflammatory cytokines [150].

Apart from their immunomodulatory effects, MSCs can preserve their stem cell characteristics and self-renewal capacity through autocrine PGE2 signaling. MSCs release PGE2 into the extracellular milieu through multidrug resistance protein 4 (MRP4), after which PGE2 binds to its receptors expressed on target cells. Activation of EP2 promotes cell proliferation and neovascularization by enhancing the secretion of vascular endothelial growth factor (VEGF). In this manner, a signaling axis involved in the regulation of the cell cycle, cellular proliferation, and cell viability is maintained [151]. In a related study, MSCs were treated with specific inhibitors to suppress PGE2 production, and cell proliferation was subsequently evaluated. It was found that inhibition of PGE2 synthesis resulted in growth arrest, whereas supplementation with MSC-derived PGE2 restored proliferative capacity [152]. Also, MSC-derived PGE2 has been identified as a central mediator of the cAMP-dependent response in both autologous and allogeneic hematopoietic stem and progenitor cell (HSPC) transplantation. MSC-secreted PGE2 selectively attenuates radiation induced apoptosis in HSPCs and significantly promotes their self-renewal capacity [153].

It has been reported that PGE2 also contributes to the migratory capacity of MSCs. Specifically, PGE2 partially enhances the migration and proliferation of human MSCs through EP2 receptor-mediated signaling involving β-arrestin-1 and c-Jun N-terminal kinase (JNK) pathways, with downstream interactions with profilin-1 (Pfn-1) and F-actin [146]. In a related study, bone marrow-derived MSCs isolated from young Wistar rats and preconditioned with PGE1 were intravenously administered via the tail vein to rats with induced pulmonary arterial hypertension. PGE1 preconditioning reduced MSC apoptosis and increased MSC migration [154].

Despite the well-established therapeutic potential of MSCs, they are increasingly recognized as having a complex and dual role in cancer pathophysiology. Within tumor microenvironments, MSCs interact with cancer cells and can contribute to tumor progression. These interactions occur not only through direct cell–cell contact but also indirectly via the secretion of cytokines, chemokines, and growth factors. In addition, metabolic mediators such as PGE2 have been implicated in MSC–tumor cell crosstalk. MSCs may also differentiate into cancer stem cells. PGE2, which induces autocrine expression of interleukin (IL)-6, IL-8, and chemokine C-X-C motif ligand-1 (CXCL1), has been shown to promote cancer stem cell formation [155,156].

### 4.2. ω-3 PUFAs and Specialized Pro-Resolving Mediators

Dietary ω-3 polyunsaturated fatty acids (PUFAs) are essential for the synthesis of cellular membranes and play an important role in maintaining the function of membrane-associated receptors. They also serve as precursors for bioactive molecules involved in the regulation of blood clotting, inflammatory responses, and vascular tone through modulation of arterial wall contraction and relaxation. In addition, ω-3 PUFAs can interact with cellular receptors that influence gene expression and activity [157]. In the context of MSC biology, ω-3 PUFAs have been shown to contribute to MSC rejuvenation through the suppression of senescence-associated gene expression. In addition, ω-3 PUFAs may promote osteoblastic differentiation via activation of Runx2 and protein kinase B (PKB/Akt) signaling pathways, while also regulating adipogenic differentiation through free fatty acid receptor 4 (FFAR4)-mediated signaling [67]. Docosahexaenoic acid (DHA) supplementation induces lipid remodeling in MSC membranes, making them resemble osteoblast plasma membrane phenotypes. This membrane reorganization enhances osteogenic differentiation of MSCs through increased activation of the Akt signaling pathway [158]. In a study conducted in mice, ω-3 PUFAs supplementation in combination with a high-fat diet was shown to be associated with reduced adipogenic differentiation and enhanced osteoblastic differentiation in BM-MSCs, along with a lower senescence phenotype and decreased osteoclast formation [159]. Another study has confirmed that ω-3 PUFAs, during rosiglitazone treatment, were shown to suppress adipogenic differentiation by downregulating adipogenesis-related genes, including adipsin and FABP4, through modulation of the peroxisome proliferator-activated receptor gamma (PPARγ)/fatty acid-binding protein 4 (FABP4) signaling axis, thereby reducing the propensity of osteocytes to undergo adipogenic conversion in mice [160]. In addition to stimulating osteoblastic differentiation in MSCs, ω-3 supplementation has also been shown to increase angiogenic and tissue regenerative potential. A study found that low-concentration ω-3 supplementation (5μM) in PL-MSCs could further enhance their already existing angiogenic potential and upregulate both basic fibroblast growth factor and vascular endothelial growth factor [161]. In a rat model, oral ω-3 PUFAs supplementation was shown to enhance the therapeutic efficacy of BM-MSCs by upregulating PPAR-γ expression. Following allogeneic transplantation in rats with unilateral ureteral obstruction, ω-3 PUFAs-conditioned MSCs exhibited stronger nephroprotective effects, including increased expression of antioxidant and anti-inflammatory markers (superoxide dismutase type 1 and IL-10) and reduced IL-6 expression and proteinuria [162].

Specialized pro-resolving mediators (SPMs) are bioactive lipid mediators derived from ω-3 PUFAs as well as arachidonic acid through enzymatic pathways involving cyclooxygenase-2 (COX-2) and lipoxygenases (ALOX-5, ALOX-12, ALOX-15). DHA is the precursor of D-series resolvins, protectins, and maresins, while eicosapentaenoic acid (EPA) gives rise to E-series resolvins, and arachidonic acid is the source of lipoxins [163]. Prostaglandins, thromboxanes, and leukotrienes initiate inflammatory responses by promoting vascular permeability, leukocyte recruitment, thrombosis, and smooth muscle contraction. In contrast, lipoxins, resolvins, protectins, and maresins act as pro-resolving mediators that suppress inflammatory signaling and facilitate apoptotic cell clearance, bacterial phagocytosis, and tissue repair [164]. Since MSCs express enzymes involved in SPM biosynthesis, their anti-inflammatory and immunoregulatory effects are thought to be partly mediated by the SPMs they produce. Accordingly, MSCs may regulate local SPM concentrations through both endogenous biosynthesis and interactions with local microenvironment [165]. In a related study, human AT-MSCs were administered via the central ear artery to male rabbits with experimentally induced gouty arthritis, resulting in increased SPM production and improved inflammation. Specifically, PGE2-mediated super-induction of SPM synthesis is the key mechanism in improving inflammation [166]. In addition, several studies have investigated the role of exogenously administered SPMs in promoting inflammation resolution by stimulating SPM production in MSCs. For example, a study investigated the immunomodulatory effects of SPMs on periodontal ligament stem cells under inflammatory conditions. IL-1β and IL-17 induced a pro-inflammatory phenotype in periodontal ligament stem cells by increasing prostaglandin-endoperoxide synthase 2 and IL-6 mRNA transcripts, whereas SPM treatment restored their pro-resolving functions, partly by upregulating 12- and 15-lipoxygenase expressions and enhancing endogenous SPM production. Furthermore, especially maresin-1, influenced the interaction between periodontal ligament stem cells and macrophages by promoting macrophages to adopt an anti-inflammatory, pro-resolving state [167]. In another study, lipopolysaccharide-stimulated human BM-MSCs were treated with resolvin E1, maresin-1, or both. At the end of the study, the group given resolving E1 and maresin-1 together showed greater activation of IL-4, IL-10, and transforming growth factor (TGF)-β1, downregulation of receptor activator of nuclear factor kappa-Β ligand (RANKL), tumor necrosis factor alpha (TNF-α), and İnterferon-gamma (IFN-γ), and a reduction in inflammation compared to the groups receiving SPM alone [168].

### 4.3. Nuclear Receptor and Lipid-Sensing Signaling

MSCs can differentiate into multiple cell types, including adipocytes, osteoblasts, chondrocytes, myoblasts, fibroblasts, and various neural-like cells. Recent findings have highlighted the role of members of the nuclear receptor superfamily in regulating MSC commitment, differentiation, and functional properties. These ligand-activated transcription factors act as molecular sensors that convert extracellular cues into specific gene expression programs, thereby directing MSC fate and function [169]. Nuclear receptors are activated by endogenous lipid-soluble ligands, including fatty acids and their metabolites, retinoic acids, oxysterols, thyroid hormones, bile acids, and steroid hormones. Specifically, PPARs (PPARα, PPAR-β/δ, and PPARγ) liver X receptor (LXR) and retinoid X receptor (RXR) are lipid-sensing nuclear receptors. Fatty acids and their enzymatically generated metabolites, including 15-HETE, 8-HETE, 15d-PGJ2, 9-HODE, 13-HODE, and leukotriene B4 (LTB4), as well as various exogenous compounds, function as potent endogenous ligands for PPARs. In contrast, oxysterols serve as ligands for the LXR/RXR receptor complex [170].

PPARγ, which is expressed in MSCs, serves as a key transcription factor regulating adipogenic differentiation and plays a critical role in directing MSC lineage commitment toward either adipocytes or osteoblasts. Reduced PPARγ expression suppresses adipocyte differentiation while promoting osteoblastic differentiation [171]. In a related study, PPARγ inhibition enhanced osteogenic differentiation of rat BM-MSCs by increasing alkaline phosphatase (ALP) activity, matrix mineralization, and the expression of osteogenic markers [172]. However, it has also been reported that lower levels of PPARγ, acting in coordination with PPARδ, may exert chondrogenic effects by promoting the differentiation of MSCs toward the chondrogenic lineage [173]. Because PPARδ has been shown to enhance osteogenic differentiation in MSCs through regulation of both lineage commitment and immunomodulatory functions [169]. A recent study demonstrated that PPARβ/δ activation in human bone marrow-derived MSCs enhanced the therapeutic effects of their conditioned medium through increased ANGPTL4 production. PPARβ/δ-modulated MSC-conditioned medium was administered intranasally to mice with endotoxin-induced acute lung injury, where it promoted lung epithelial repair, improved endothelial barrier integrity, and reduced pro-inflammatory cytokine release [174]. However, PPARα appears to have a limited role in the direct regulation of MSC differentiation [169].

Another nuclear receptor that stimulates adipogenic differentiation is RXR. However, RXR activation may be responsible for the development of more dysfunctional adipose tissue. A study compared adipocytes transformed from MSCs under the conditions of PPARγ activator (rosiglitazone), RXR activator (IRX4204), and the endocrine disruptor tributyltin. The study concluded that RXR activators (tributyltin and IRX4204) produced more dysfunctional fat cells compared to rosiglitazone [175]. It has also been shown that RXR activation can lead to inhibition of osteogenesis [176]. In a related study noted that all agonists (tributyltin, rosiglitazone, RXR agonists) can suppress osteoblast differentiation, reducing mineralization, alkaline phosphatase activity, and gene expression related to bone formation [177].

**Figure 2:**
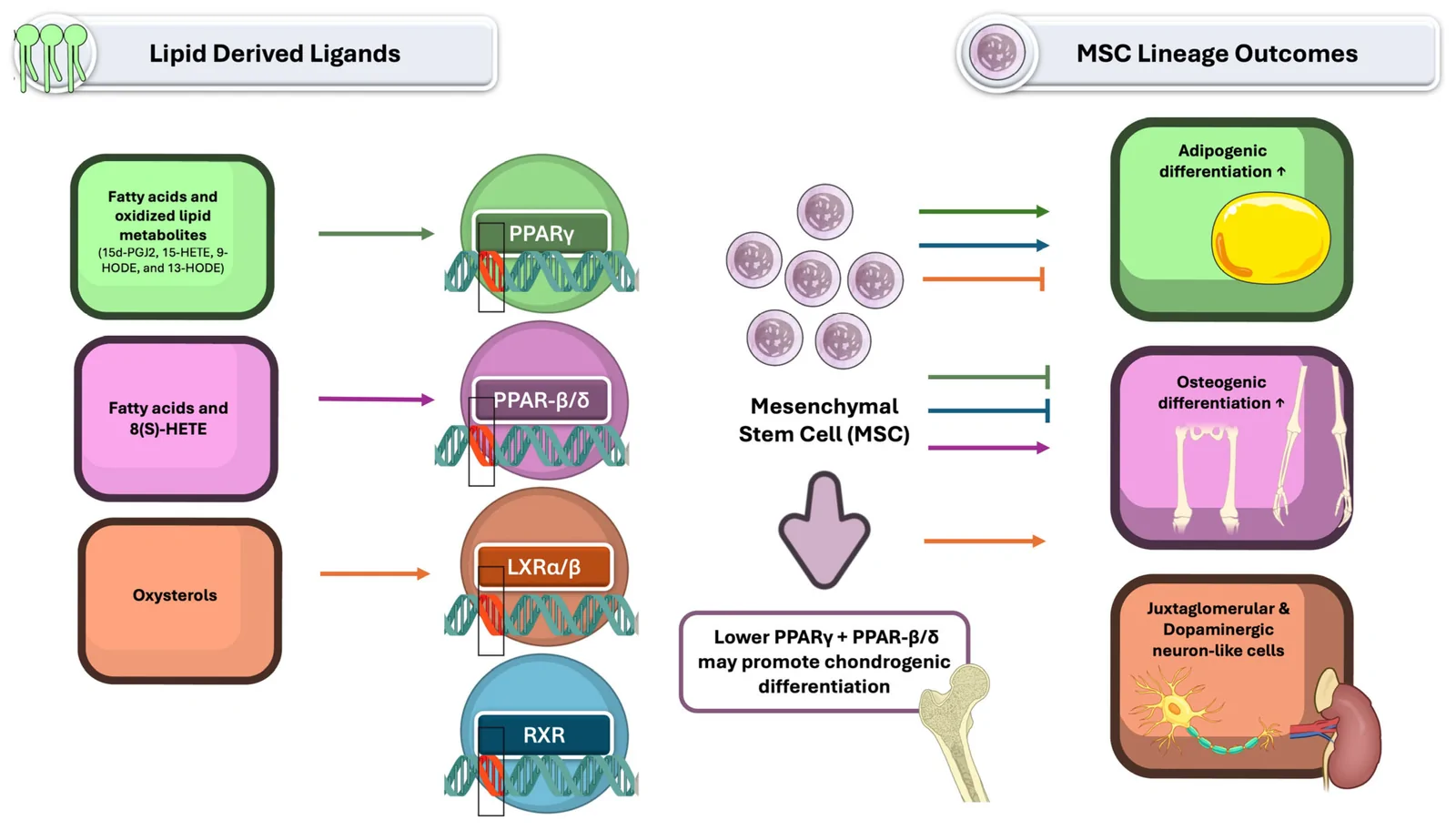
Role of lipid-activated nuclear receptors in MSC lineage commitment [169,170]. PPARγ: Peroxisome proliferator-activated receptor gamma, PPAR-β/δ: Peroxisome proliferator-activated receptor beta/delta, LXRα/β: Liver X receptor alpha/beta, RXR: Retinoid X receptor, 15d-PGJ2: 15-deoxy-Δ12,14-prostaglandin J2, 15-HETE: 15-Hydroxyeicosatetraenoic acid, 9-HODE: 9-Hydroxyoctadecadienoic acid, 13-HODE: 13-Hydroxyoctadecadienoic acid, 8(S)-HETE: 8(S)-Hydroxy eicosatetraenoic acid. Green, purple, orange and blue arrows represent PPARγ-, PPAR-β/δ-, LXRα/β- and RXR-mediated effects, re-spectively. Upward arrows (↑) indicate an increase in the indicated differentiation outcome, while arrowheads (→) indicate a stimulatory effect and T-shaped ends (⊣) indicate an inhibitory effect.

LXR has also been implicated in the regulation of adipogenic differentiation in MSCs. While deletion of LXRα promotes adipogenesis, its overexpression suppresses adipogenic differentiation, potentially through modulation of the Wnt/β-catenin signaling pathway [178]. In a study using a mouse model of corticosterone-induced Cushing’s syndrome, intraperitoneal administration of tonsil-derived MSCs was shown to reduce circulating LDL cholesterol levels and promote weight loss through downregulation of LXRα [179]. Furthermore, expression of the LXR in MSCs has also been studied in the context of their lineage differentiation potential toward other cell types. A study showed that LXRα, when activated by its ligands or cAMP, increased renin gene expression in both mouse and human MSCs. Under sustained cAMP stimulation and with LXRα overexpression, murine MSCs differentiated into juxtaglomerular cell-like cells [180]. Another study found that combined treatment of rat BM-MSCs with growth factor and LXR agonist increased LXRα and LXRβ protein expression and transformed the MSCs into dopaminergic neuron-like cells that express tyrosine hydroxylase [181]. Role of lipid-activated nuclear receptors in MSC lineage commitment was illustrated in Figure 2.

## 5. Cellular and Molecular Mechanisms of Lipid-Driven MSC Regulation

### 5.1. Lipids and Asymmetric Cell Division in MSCs

MSCs preserve their self-renewal capacity through asymmetric cell division, a mechanism essential for tissue development and homeostatic maintenance. During this process, a single mitotic event generates two daughter cells with distinct destinies: one retains stem cell properties, whereas the other undergoes differentiation into a specialized non-stem cell type. Stem cells can also undergo symmetric cell division, producing two identical daughter cells that share the same cellular characteristics and fate [182]. Lipids play an active regulatory role in cell division. During cell division, certain membrane phosphoinositides contribute to the correct positioning of the mitotic spindle, thereby regulating the axis of cell division. During cytokinesis, lipids such as phosphoinositides, sphingomyelin, and ganglioside GM1 are required for successful cell cleavage and separation. Furthermore, enrichment of PE during the final stage of division is essential for the complete separation of daughter cells [183]. Free fatty acid oxidation plays a role in energy production as well as in directing cell division. Activation of the PPAR-δ signaling pathway increases the oxidation of free fatty acids, which may promote asymmetric division of MSCs and thereby contribute to the preservation of the MSC pool. In contrast, a reduction in free fatty acid oxidation may shift MSCs toward symmetric division [184]. In addition, acetyl-CoA generated during fatty acid oxidation is essential for histone acetylation, which may regulate genes involved in asymmetric division and differentiation. Therefore, lipid metabolism may indirectly contribute to MSC division and fate determination through epigenetic and polarity-associated mechanisms [26].

### 5.2. Lipid-Dependent MSC Niche Interactions

The stem cell niche constitutes a specialized microenvironment that provides both structural support and regulatory signals required for stem cell maintenance and appropriate differentiation responses. This niche is composed of cellular elements, including neighboring supportive cells, immune cells, and parenchymal cells, as well as non-cellular components such as the extracellular matrix (ECM), signaling mediators (e.g., growth factors, cytokines, and extracellular vesicles), and external environmental factors [185]. Among external environmental factors, obesity and high-fat diet–associated alterations in the bone marrow microenvironment represent important regulators of MSC fate. Increased bone marrow adiposity and the development of leptin resistance in bone marrow MSCs promote PPARγ-mediated adipogenesis while suppressing osteogenesis [186]. Increased bone marrow adiposity can also skew myeloid-prone hematopoietic stem cell differentiation and mimic aging-like effects, while concurrently biasing resident mesenchymal/skeletal-lineage stromal cells toward the adipogenic fate at the expense of the osteogenic and hematopoiesis-supportive compartments [187]. The immunomodulatory properties of MSCs are also highly influenced by external environmental factors. For example, palmitate has been reported to alter the immunomodulatory effects of MSCs on macrophages through activation of the ceramide/CCL2 signaling pathway [188]. Sphingosine-1-phosphate (S1P) is a bioactive lipid signaling mediator involved in intercellular communication through activation of specific S1P receptors. Through the regulation of ECM–cytoskeleton organization, S1P can modulate MSC activation, migration, and tissue regeneration, thereby contributing to the dynamic regulation of the stem cell niche [189].

Lipid and lipid mediators secreted by cells within the stem cell niche can also modulate MSC activity and function. Fatty acids released from bone marrow adipocytes can serve as an important metabolic energy source for highly proliferative hematopoietic cells. Adipocyte-derived fatty acids have been reported to support hematopoietic stem cell recovery under stress conditions and to promote the function of stem cells [190]. Osteoblasts within the bone marrow niche contribute to the regulation of hematopoietic stem cells through the production of PGE2. PGE2 interacts with specific receptors expressed on the surface of HSCs, leading to activation of the Wnt signaling pathway, which plays a critical role in HSC specification and development [26].

### 5.3. Lipid Raft Dynamics and Membrane Signaling Platforms

Lipid rafts are highly dynamic membrane microdomains enriched in cholesterol and sphingolipids that play essential roles in numerous biological functions. Upon specific stimuli, these domains can cluster together and act as signaling platforms for the assembly of protein complexes, thereby promoting cellular communication and interactions with the extracellular environment [191]. The dynamic behavior of lipid rafts is also thought to play an important role in regulating signaling pathways essential for stem cell function [26]. Much of the mechanistic detail in this regard derives from hematopoietic stem cells, in which lipid raft-dependent receptor clustering has been studied most extensively; these findings are summarized here as a conceptual framework that is increasingly, though not yet fully, extrapolated to mesenchymal stromal/stem cells, where direct evidence remains comparatively limited. For example, in hematopoietic stem cells, certain cell surface receptors and signaling molecules assemble within lipid raft domains, thereby facilitating the recognition of the surrounding microenvironment, maintenance within the niche, migration, mobilization from the bone marrow into circulation, and homing to target tissues [192]. The interaction between very late antigen-4 (VLA-4) and C-X-C chemokine receptor type 4 (CXCR4) on hematopoietic stem cells, and vascular cell adhesion molecule-1 (VCAM-1) and stromal cell-derived factor-1 (SDF-1) within the bone marrow niche, maintains the retention of hematopoietic stem cells in the bone marrow microenvironment. In contrast, phospholipase C beta 2 (PLC-β2)- and membrane-type 1 matrix metalloproteinase (MT1-MMP)-mediated mechanisms disrupt lipid raft integrity, thereby weakening these adhesion interactions and ultimately promoting their mobilization into the circulation. When signals such as stem cell factor (SCF), IL-3, IL-6, and VEGF are present, increased lipid raft clustering, recruitment of the cluster of differentiation 117 (CD117) receptor into lipid rafts, and activation of the phosphoinositide 3-kinase (PI3K)–protein kinase B (Akt)–forkhead box O (FOXO) signaling pathway collectively promote the proliferation of hematopoietic stem cells [193]. In MSC specifically, the prion protein is also consistently found in lipid raft microdomains, and prion protein has been reported to support cellular proliferation and facilitate self-renewal in human MSCs [194]. Furthermore, netrin-1 enhances human umbilical cord blood-derived MSC proliferation and wound healing by promoting integrin α6β4/c-Src-mediated recruitment of signaling molecules into lipid rafts [195].

Lipid rafts also regulate MSC differentiation through dynamic signaling platform reorganization. In a related study, it has been shown that the relocalization of ATP6AP2 within lipid rafts critically regulates the neuronal differentiation of adipose-derived MSCs by modulating Wnt signaling balance, G protein switching, and downstream extracellular signal-regulated kinase (ERK)/c-Jun N-terminal kinase (JNK) pathway activation [196]. Another study has shown that disruption of lipid raft integrity using methyl-β-cyclodextrin suppresses lineage-specific gene expression, thereby blocking osteogenic, chondrogenic, and adipogenic differentiation in human dental pulp-derived stem cells. This study indicating that intact lipid rafts are essential for multilineage differentiation [197].

### 5.4. Lipid Regulation of the Transcriptome and Epigenome

Multipotent MSCs undergo a tightly regulated and complex gene expression program during their differentiation. Transcriptomic approaches can be utilized to elucidate the molecular mechanisms underlying MSC differentiation [198]. Consequently, lipids and lipid metabolic pathways may regulate stem cell behavior by modulating the expression of stem cell-specific genes, either through direct transcriptional regulation [26]. For example, integrated transcriptomic and lipidomic analyses in human MSCs demonstrated that anti-aging compounds, particularly metformin, modulate phospholipid and fatty acid metabolism while enhancing trilineage differentiation capacity. Specifically, the biosynthesis of PCs and PEs was suppressed, whereas phosphatidylinositol production and the conversion of saturated fatty acids to monounsaturated fatty acids were enhanced, suggesting that lipid remodeling contributes to the regulation of MSC senescence and differentiation programs [199]. Similarly, integrated metabolomic and transcriptomic analyses in rat bone marrow MSCs revealed that cellular senescence is strongly associated with alterations in lipid metabolism pathways. Several lipid metabolism-related genes, such as stearoyl-CoA desaturase 2 (SCD2), fatty acid desaturase 2 (Fads2), and diacylglycerol O-acyltransferase 2 (Dgat2), were identified as key regulators of MSC aging, while Scd2 overexpression alleviated cellular senescence in aged MSCs [200].

Epigenetic modifications play a crucial role in regulating gene expression and the cellular differentiation of MSCs by directly altering DNA and histone structures. In addition, microRNAs (miRNAs), although not directly modifying chromatin, regulate MSC differentiation indirectly by targeting key epigenetic enzymes [201]. Lipids regulate gene expression involves the modulation of epigenetic modifications [26]. However, examples of epigenetic regulation by lipids in MSCs are limited. Studies have mostly evaluated the role of an obesogenic environment, created by a high-fat diet, in epigenetic regulation in MSCs. In a related study, it was shown that obesity induces epigenetic alterations in swine MSCs, including changes in DNA hydroxymethylation and histone methylation patterns, which are associated with impaired MSC migration and proliferation [202]. Moreover, obesity has been associated with widespread alterations in DNA hydroxymethylation patterns in swine and human adipose-derived MSCs, particularly in genes involved in apoptosis, cell proliferation, and cellular senescence [203]. In a study investigating 5-hydroxymethylation changes in mitochondria-related genes in MSCs obtained from pigs with metabolic syndrome induced by a high-fat diet, increased 5-hydroxymethylation levels were observed particularly in genes involved in fatty acid metabolism, whereas decreased 5-hydroxymethylation levels were detected in genes associated with electron transport chain activity and energy production [204]. Obesity has also been shown to induce epigenetic alterations in mitochondria-related genes in human adipose-derived MSCs, accompanied by impaired mitochondrial structure and function, increased oxidative stress, disrupted fatty acid metabolism, and reduced neurogenic differentiation capacity [205]. Another study showed that UC-MSCs obtained from infants of obese mothers exhibited increased lipid accumulation, reduced fatty acid oxidation, and dysregulated AMP-activated protein kinase (AMPK) activity during in vitro myogenesis. Specifically, DNA methylation analysis revealed hypermethylation accompanied by decreased mRNA expression of genes involved in fatty acid oxidation [206]. Collectively, these studies suggest that lipids and obesity-associated metabolic disturbances may reprogram stem cell behavior through epigenetic mechanisms. Alterations in DNA methylation and hydroxymethylation of genes involved in lipid metabolism and energy production appear to play a regulatory role in MSC metabolic profile, mitochondrial function, and functional properties.

## 6. Diet-Driven Molecular Remodeling of Mesenchymal Stem Cells

Because functional MSC number is regulated by the interactions between genetic and epigenetic factors such as environmental exposures and diet, diet, aging, inflammation, and microbiota play a significant role in maintaining the health, tissue function, and regenerative capacity of MSCs in various tissues [207]. MSCs are affected by their local environment, the stem cell niche. The nutritional medium has been shown to influence the number and quality of stem cells, ensuring their suitability for regeneration and tissue repair processes [208]. Therefore, determining the nutritional requirements of stem cells and understanding the effect of diet on the quantity and quality of stem cells is crucial for supporting the healing of damaged tissues and cell regeneration [209]. Many studies have investigated how diet affects the behavior of adult stem cells and has shown links between food products, the microbiome system, and stem cells. However, more research is needed to establish clear guidelines and evidence-based protocols for successfully integrating such interventions into stem cell therapy [210,211].

### 6.1. High-Fat Diet and Obesity-Associated MSC Dysfunction

Adipose tissue was identified as a novel and important source for MSCs when stromal vascular cells, first discovered in rats in the 1960s, were later found to exhibit multipotent properties in humans as well [212,213]. ASCs directly contribute to homeostasis, cell regeneration, and spontaneous repair of adipose tissue [214]. Compared to other MSCs, ASCs demonstrate clear advantages due to their accessibility, lack of ethical concerns, available sample quantity, ease of in vitro isolation and propagation, and the possibility of having an autologous source [215].

Adipose tissue is highly heterogeneous in terms of its composition and distribution throughout the body. Subcutaneous white adipose tissue (scWAT), located beneath the skin, is the largest storage area for excess lipid [216]. The expansion of scWAT is determined by two main processes: the differentiation of new mature fat cells from their precursors known as MSCs, and their ability to grow from already formed fat cells [217]. When scWAT reaches its maximum storage capacity, adipose tissue can no longer properly store lipids and redirects this lipid flow to other organs where it accumulates as ectopic fat, causing lipotoxicity and inflammation [218]. This ectopic accumulation occurs primarily in visceral adipose tissue (vWAT) and the liver, leading to visceral obesity and hepatic steatosis [219].

Obesity is a complex disease resulting from factors such as excessive food intake, impaired basal metabolism, and low energy expenditure; however, genetic and epigenetic factors also play a role. White adipose tissue (WAT) dysfunction is the primary consequence of obesity [220]. The changes in WAT function caused by obesity also affect stem cells located in adipose tissue; these cells include MSCs, a heterogeneous population consisting of stromal cells, progenitor cells, fibroblasts, and stem cells. Given the role of MSCs in bone, cartilage, and adipose tissue regeneration and in the body’s homeostasis, impaired MSC activity has significant health consequences [221].

The effect of obesity on adipogenic plasticity is represented in the literature by two different dysfunctional orientations: some studies argue that increased pro-inflammatory cytokines and PPARγ/Sirtuin 1 (SIRT1) suppression blunt functional differentiation [222,223,224]; other studies show that cells acquire a pathological phenotype by entering an early adipocyte orientation through overactivation of the PI3K/Akt/mTOR pathways [225,226]. In particular, a high-fat diet (HFD) weakens the clinical efficacy of MSCs by transforming them from a regenerative source into a pro-inflammatory and functionally impaired cell population [227].

In addition to ASCs, obesity also compromises the functionality of other MSCs. Specifically, HFD misprograms BMSCs, directing them towards pathological fat cell production via the JAK2/STAT3 signaling pathway instead of bone repair, and weakening the body’s blood-forming capacity [186,228,229]. This molecular disruption also has an intergenerational effect; maternal obesity alters genetic codes such as AMPK activity and DNA methylation in the cord blood stem cells of infants, causing the risk of obesity to be passed on to the next generation at birth [206,230]. In conclusion, obesity damages not only fat tissue but also the entire stem cell system in the body, causing these cells to lose their healing power and laying the groundwork for many chronic diseases [231].

*Evidence from animal models*

Previous studies have shown that HFD-induced obesity significantly impairs the function of MSCs obtained from bone marrow and visceral and subcutaneous adipose tissue. MSCs from visceral white adipose tissue (vWAT) have been reported to have reduced proliferation capacity and increased cellular senescence. These changes have been suggested to be associated with impaired paracrine functions, such as the detoxification response to toxic substances and drugs [232,233]. Since increased visceral fat tissue raises the risk of cardiovascular disease and type 2 diabetes, these findings are important in explaining the negative effects of obesity on health [234]. These results are consistent with other studies reporting that obesity increases oxidative stress and endoplasmic reticulum stress by altering the autophagy/mitophagy balance, which in turn contributes to MSC dysfunction and cellular senescence [235,236].

The increasing prevalence of high-fat diets (HFDs) (≥30% of energy from fat) is raising concerns about their potential negative impacts on health, particularly obesity and other metabolic comorbidities [237,238]. In general, HFD increases glucose availability but does not necessarily increase its utilization; it increases circulating levels of certain types of unsaturated fatty acids that can act as ligands for various signaling pathways; and it causes insulin resistance [239].

Studies using current lipidomic analysis have found significant differences in the formation of lipid types and ratios in the tissues of animals fed hypercaloric diets compared to animals fed normal diets. One study reported increased levels of diacylglycerol and PC in rats fed an obesogenic diet [240]; another study reported that triglyceride (TG) accumulation in tissues other than adipose tissue is a reflection of lipotoxicity [241]. Studies have shown that certain lipid classes, including glycerolipids, glycerophospholipids, sphingolipids, and fatty acids, as well as TG in visceral adipose tissue, were dysregulated in rats fed HFD compared to normally fed controls [242].

Experimental animal models support the effect of obesity-inducing diets on MSCs. In a pig model created with a high-carbohydrate and cholesterol-rich diet, various features of metabolic syndrome were observed, including abdominal obesity, hypertension, hypertriglyceridemia, and insulin resistance. Importantly, MSCs isolated from obese pigs exhibited increased adipogenic differentiation and increased cellular senescence, suggesting that obesity-related metabolic stress can impair stem cell regenerative potential [243,244]. One study identified proteins related to the leptin signaling pathway in the MSCs of mice fed a normal diet and showed that these molecules were lost in obese mice induced by HFD [245]. Similarly, the literature on the role of leptin in lipid homeostasis and energy balance has shown that HFD causes a decrease in leptin expression levels in MSCs [246,247,248]. Leptin deficiency can cause obesity, and obesity can also lead to a decrease in leptin expression levels [249,250]. A study involving gene expression analysis showed that, compared to a normal diet, HFD-fed MSCs obtained from adipose tissue resulted in a significant upregulation of SCD1. This overexpression appears likely to be due to leptin deficiency, as leptin is known to inhibit SCD1 at the transcriptional level [251]. SCD1 plays a role in ion binding, lipid oxidation, oxidoreductase activity, and stearoyl-CoA 9-desaturase activity, which links it to metabolic disorders such as obesity and insulin resistance [246]. Additionally, HFD was found to cause a significant decrease in the expression of SOX2 and KLF4 genes, which are key transcription factors involved in regulating cellular self-renewal, development, and differentiation processes [252,253]. Decreased expression of SOX2 and KLF4, these stem cell-related factors, can impair the regenerative capacity and differentiation potential of MSCs in obese individuals, as reported in the literature [254]. In addition, HFD feeding has also been shown to suppress the Wnt/β-catenin signaling pathway, which plays a critical role in MSC regeneration, differentiation, and homeostasis [255]. Decreased Wnt signaling has been associated with increased senescence of MSCs, decreased stem cell capacity, and increased adipocyte differentiation [256,257].

*Evidence from in vitro lipid exposure studies*

HFD has been shown to affect stem cell function in a tissue-specific manner. In lung stem cells, HFD increased stem cell activity, whereas switching to a low-fat diet reversed several of these HFD-induced effects [258]. The effects of HFD on stem cells appear to be reversible and therefore can be corrected with dietary changes alone. While some studies report that HFD disrupts neurogenesis [259,260], certain types of fatty acids, including omega-3 fatty acids, appear to enhance neurogenesis [261,262]. This suggests that diet could be used to compensate for neuronal loss in diseases and aging. In general, it is necessary to investigate whether the differences observed after HFD are related to specific changes in lipid homeostasis and whether the changes in stem cell behavior are a direct consequence of these changes in lipid homeostasis [26].

In this context, lipid metabolism disorders caused by obesity and high-fat diets are thought to play an important decisive role in the regenerative capacity, differentiation potential, and cellular homeostasis of MSCs. Specifically, changes in lipid homeostasis may affect stem cell behavior, contributing to MSC dysfunction and the development of chronic diseases.

### 6.2. Caloric Restriction (CR) and Metabolic Optimization

Caloric restriction is defined as either reducing calorie intake by more than 20% compared to unrestricted eating, or increasing the time between meals [263]. This process involves several systemic changes, primarily a decrease in glucose availability and oxidation, an increase in lipid utilization, and the production of ketone bodies as alternative carbon sources [239]. CR has been shown to extend lifespan and reduce the risk of developing age-related diseases in many species, from yeast to primates [226]. Therefore, CR is considered a promising strategy that supports healthy aging and improves metabolic health [264]. CR is a non-genetic dietary intervention that has been reported to support stem cell maintenance and survival by modulating cellular energy metabolism, thereby potentially providing a favorable microenvironment for stem cell survival [265].

The positive effects of CR on metabolic health are primarily based on its ability to reorganize energy metabolism. Specifically, it reduces WAT mass, increasing fatty acid oxidation and preventing ectopic fat accumulation. Furthermore, by increasing insulin sensitivity and improving glucose tolerance, it contributes to maintaining metabolic homeostasis [266]. Long-term CR has been reported to cause a reduction in adipocyte size and a positive redistribution of body fat. During this process, a shift from vWAT to scWAT occurs, a change considered significant for aging and metabolic health. While scWAT exhibits more protective metabolic properties, vWAT is associated with obesity and age-related adverse health outcomes. Therefore, this redistribution contributes to an extended healthy lifespan and improved quality of life [267]. However, these effects may not be uniform across tissues or physiological conditions.

Caloric restriction (CR) has been shown to improve SIRT1 activity in skeletal muscle by increasing both nicotinamide adenine dinucleotide (NAD^+^) expression and cellular levels [268,269]. SIRT1 supports the preservation of dormant MSCs and the survival of differentiated cells by suppressing pathways associated with apoptosis and inflammation. CR-induced metabolic adaptations may also alter MSC differentiation and cellular resource allocation, thereby influencing tissue regeneration [270,271,272]. Under restrictive dietary conditions, these adaptations may favor the allocation of limited cellular resources toward tissue maintenance and regeneration [273]. Altered phospholipid composition in plasma and tissues, particularly PC and PE levels, may indicate impaired membrane integrity and cellular signaling pathways [274]. These changes, along with elevated ceramide levels and an imbalance between saturated and unsaturated fatty acids, can exacerbate insulin resistance and promote inflammatory responses in affected tissues [275,276,277].

Studies have shown that long-term CR reduces age-related adverse changes in adipocytes and ASCs, increases autophagy to decrease senescent cell accumulation, and supports stem cell characteristics. Protective effects on age-related processes such as insulin resistance, inflammation, and tissue fibrosis have also been reported [278]. Short-term CR has been reported to reduce body weight, fat mass, and glucose levels; it has also been shown to decrease adipocyte size and inflammatory gene expression [266,279]. Based on this information, it is predicted that CR, depending on its duration, can modulate adipose tissue and stem cell biology, thereby having significant effects on metabolic health and regenerative capacity. Importantly, the effects observed in aging models or metabolically impaired conditions may not necessarily be extrapolated to younger or metabolically healthy individuals.

Furthermore, the literature has shown that bariatric surgery, a form of CR (which can result in substantial reductions in energy intake), can enhance stem cell characteristics in ASCs. This effect is reportedly related to the induction of autophagy processes and the suppression of adipogenic differentiation [226,280]. These findings suggest that CR can support regenerative capacity and may play a significant role, particularly in preserving the function of ASCs. This presents significant potential for age-related tissue damage repair and regenerative medicine applications [267].

Caloric restriction (CR) is considered a biological stressor because it regulates energy and nutrient sensing pathways as well as stress resistance signals. This process activates key regulators such as AMPK, mTOR, Nrf2, SIRT-1, FOXO, and PGC-1α, which are involved in longevity processes such as autophagy, mitochondrial biogenesis, DNA repair, and the expression of antioxidant and detoxifying enzymes. While mild to moderate levels of biological stress can provide significant health benefits, higher stress intensities can be harmful to cells; therefore, such anti-aging strategies need to be closely monitored to achieve health gains [281].

In summary, while the metabolic effects of different dietary models, such as pure calorie restriction, prolonged fasting, ketogenic diets, high-fat diets, and Western-style diets, on the organism differ considerably, these interventions may converge on common metabolic adaptations that influence the self-renewal capacity of stem cells in many tissues. Although the mechanisms underlying these responses are not yet fully understood, several common metabolic features have been proposed, including increased fat utilization accompanied by decreased glucose availability, direct effects of dietary patterns on intracellular metabolic processes in stem cells, metabolic adaptations induced by changes in fasting and satiety states, and activation of shared metabolic stress pathways. These mechanisms may represent a common adaptive response of stem cells to changes in nutrient availability and contribute to the maintenance or expansion of the stem cell pool [239].

In this context, caloric restriction may play a role in protecting MSCs by reprogramming energy metabolism, maintaining their regenerative capacity, and reducing age-related cellular damage; in particular, CR-activated SIRT1, AMPK, and autophagy-related pathways may exert protective effects on metabolic health and tissue regeneration by supporting stem cell homeostasis. Differences in diet duration, age, metabolic status, tissue origin, and experimental model may contribute to the heterogeneous findings reported in the literature. Therefore, further studies are needed to determine the conditions under which CR exerts beneficial or detrimental effects on MSC lipid metabolism and function.

### 6.3. Dietary Patterns and Bioactive Nutrients

Nutrition and lifestyle directly affect the body’s regenerative capacity by regulating the quantity, quality, and niche (microenvironment) of stem cells. Meeting the specific metabolic needs of stem cells is crucial for slowing down deterioration in biological systems and reducing the burden of disease. Therefore, changes in the nutritional environment determine the fate of stem cells; inadequate or unbalanced nutrition restricts the repair potential of these cells, paving the way for premature aging and systemic dysfunction [208].

Stem cells function as a dynamic system that translates dietary inputs into cellular decisions. Nutrients can directly influence stem cell behavior by acting on cellular metabolic and signaling pathways or indirectly influence stem cells by regulating the stem cell niche or modulating systemic hormone production [282]. In response to these stimuli, stem cells activate signaling pathways, reprogram their metabolism and gene expression, and transform nutritional inputs into processes such as differentiation and self-renewal. Fundamental characteristics of stem cells, such as their division balance, genomic integrity, autophagy processes, and differentiation capacity, are shaped by nutrient availability. Mechanistically, this delicate balance is controlled through key regulators like mTORC1, which sense nutrients and manage both metabolism and stem cell fate [283]. In this context, dietary models and bioactive components play a critical role in maintaining tissue repair capacity by preventing stem cell depletion.

Amino acids (AAs) play a crucial role in maintaining the pluripotent capacity, self-renewal, and differentiation abilities of stem cells [284]. The presence of essential amino acids, in particular, enhances proliferation while preserving stem cell characteristics [285,286]. In this process, the mTORC1 cascade, which acts as the primary nutrient sensor, is the key mechanism that controls stem cell proliferation and autophagy by regulating protein synthesis and growth [287,288]. Restricting or supplementing specific amino acids in the diet can directly alter stem cell fate. For example, methionine deficiency suppresses cell proliferation [289,290], while arginine promotes tissue regeneration [291].

Fatty acids (FAs) possess a specific lipidome signature that governs essential physiological processes in MSCs, such as restorative balance, self-renewal, and symmetric-asymmetric division. The delicate balance between fatty acid synthesis and fatty acid oxidation in MSCs is crucial for preventing cellular depletion [26]. This research demonstrates that reduced saturated fatty acid oxidation can decrease human BMSC proliferation and lead to cell death, suggesting that saturated fatty acids may play a role in the long-term impairment of in vivo BMSC survival [134]. Conditions such as obesity or HFD disrupt this balance, pushing MSCs more towards adipogenic tendencies [227]. Alpha-linolenic acid (ALA), EPA, and docosahexaenoic acid (DHA), which are ω-3 series fatty acids, and linoleic acid (LA), γ-linolenic acid (GLA), and arachidonic acid (ARA), which are ω-6 series fatty acids, have different biological effects on MSCs [292]. ARA has been shown to increase fat cell formation (adipogenesis) in human MSC cultures and to inhibit bone-forming cell formation (osteoblastogenesis) at high concentrations. In MSCs, arachidonic acid from the ω-6 series promotes adipocyte differentiation while suppressing osteoblast differentiation. In contrast, ω-3 series fatty acids have not had a significant effect on adipogenic differentiation; their effects on osteoblastogenesis have been reported to be more limited [293].

Dietary habits and biologically active nutrients are among the key factors determining stem cell performance and tissue balance in the body. Current research reveals that micronutrients not only support cellular metabolism but can also directly modulate signaling pathways and cellular processes in MSCs, thereby influencing stem cell activity. Vitamins, minerals, and phytochemicals, in particular, play a critical role in maintaining the proliferation and self-renewal capacity of stem cells by influencing gene expression and epigenetic regulation [16]. However, some nutritional effects on MSCs may also occur indirectly through systemic metabolic changes or alterations in the tissue microenvironment. The emergence of nutritional regulation as a target for stem cell function suggests that these substances can influence not only cell survival but also the sustainability of stem cell pools in tissues. In this context, a direct link has been identified between the influence of specific molecules and stem cell-mediated tissue homeostasis and differentiation; in particular, vitamins A, D, C, E, and B group vitamins, as well as minerals such as zinc, selenium, magnesium, and calcium, stand out as key micronutrients for stem cell function [294,295,296,297].

Vitamin A, derived from carotenoids, is a powerful regulator of MSC renewal and differentiation processes at both genetic and epigenetic levels [298]. Vitamin A can modulate antioxidant and anti-inflammatory pathways by regulating key effectors such as SIRT1, Wnt, NF-κB, and Nrf2 [299].

Vitamin C (ascorbic acid) is a well-known promoter of MSC proliferation and self-renewal [300,301]. This vitamin can accelerate cell population doubling and preserve stem cell characteristics of MSCs by increasing the expression of genes related to versatility [302,303]. Conversely, excessive concentrations can inhibit cell growth and even trigger apoptosis [302].

Vitamin D (1,25-dihydroxyvitamin D3) also has a complex regulatory effect on the fate of MSCs. Its active metabolite has been reported to support the maintenance of stem cell characteristics by increasing the expression of versatility markers and simultaneously reducing the prevalence of senescent cells [304,305]. The effects of vitamin D are highly dose dependent; both deficiency and oversupply lead to suppression of MSC proliferation [306]. Other lipid-soluble vitamins, including vitamin E and K_2_ may act synergistically with vitamin D to promote the differentiation of MSCs toward the osteogenic lineage and enhance the expression of key osteogenic transcription factors. Vitamin E may additionally regulate MSC proliferation and cell survival by modulating signaling pathways, further supporting its role in MSC growth and differentiation [303,307].

B vitamins are of fundamental importance as cofactor enzymes that catalyze essential metabolic pathways. Vitamins B6, B9, and B12, in particular, are an integral part of one-carbon metabolism and therefore influence epigenetic mechanisms such as DNA and histone methylation, and are thus crucial for self-renewal processes and lineage commitment [308,309]. In addition, nicotinamide (vitamin B3) has been shown to delay replicative senescence in MSCs [310].

In addition to vitamins, various trace elements are critical for maintaining the functional integrity of MSCs. Magnesium has been shown to support MSC viability and proliferation in a dose-dependent manner [311,312,313,314]. Magnesium deficiency can accelerate cellular senescence and stem cell depletion, while excessively high concentrations can be inhibitory or cytotoxic [315].

Similarly, zinc is crucial for the maintenance of MSCs, as optimal levels promote proliferation and increase the expression of essential versatility and telomerase-related genes, while high concentrations are cytotoxic [316]. Selenium also has a dual effect; at low concentrations it supports MSC viability and mitigates aging, but at higher levels it becomes harmful [313,317].

Inadequate or abnormal calcium intake early in life can reprogram the MSC differentiation capacity of male offspring, exacerbating high-fat diet-related obesity in adulthood. This finding represents an indirect nutritional effect on MSCs, as early-life nutritional exposure may alter systemic metabolic and developmental conditions that subsequently shape the MSC niche. This process is particularly associated with the suppression of the Wnt/β-catenin signaling pathway [318]. Adipogenic differentiation of MSCs is regulated by complex networks such as JAK2/STAT3, SIRT1/SIRT2, ERK1/ERK2, and TGF-β/BMP, while activation of Wnt/β-catenin signaling inhibits adipogenic differentiation and promotes osteogenic differentiation [86,319]. Since the differentiation potential of MSCs decreases significantly with age, nutritional status during pregnancy and lactation, as well as exposure to adverse factors, determine the fate of MSCs, thus laying the foundation for metabolic disorders in adulthood [86,320]. Furthermore, the presence of calcium (Ca^2+^) ions in the microenvironment exhibits osteo-inductive properties, directly supporting the migration of MSCs to bone tissue [321].

Natural bioactive compounds found in seaweed, grasses, fruits, and vegetables can regulate the self-renewal and differentiation potential of adult stem cells, primarily through intracellular signaling pathways [8,322,323]. Although bioactive components have not been widely used in the clinic due to their variability and complexity, an increasing number of studies have focused on the modifying effects of natural compounds on MSCs [8].

Phytochemicals possess antioxidant, anti-inflammatory, mitochondrial protective, and metabolism -activating properties, and also influence key signaling pathways related to the survival, differentiation, migration, and regenerative capacities of MSCs [16]. They are also vital in stem cell physiology through their roles in cell signaling, cell cycle regulation, oxidative stress response, and inflammation processes [324]. Among phytochemicals, polyphenols in particular may directly modulate intracellular signaling and oxidative stress responses in MSCs, while their effects may also occur indirectly by regulating the microenvironmental niche [325,326]. Phytochemicals significantly influence proliferation and differentiation and accurately regulate MSCs through various protein pathways. Regular intake of phytochemicals such as curcumin, EGCG, resveratrol, allicin, coenzyme Q10, and melatonin can significantly enhance the effectiveness of regenerative therapies involving MSCs. Curcumin and EGCG, as well as curcumin and coenzyme Q10, have shown the potential to increase the resistance of MSCs to oxidative stress, chronic inflammation, and tissue damage [327,328,329]. The main vitamins, minerals, and phytochemicals that affect MSC function are summarized in Table 2.

Although there is little direct clinical evidence linking micronutrient concentrations to the effectiveness of MSC therapy, data from hematopoietic stem cell transplantation suggest that common vitamin and mineral deficiencies can affect healing, immune function, and worsen clinical outcomes in patients [330,331]. These findings underscore the potential role of fortified foods and personalized nutrition strategies in supporting micronutrient availability in humans and creating a favorable environment for the survival, reproduction, and differentiation of MSCs [16]. These regulatory mechanisms play a critical role in maintaining the metabolic health of MSCs and sustaining their tissue regeneration capacity. While current evidence suggests that specific dietary strategies can improve cellular metabolism via epigenetic regulation and redox-inflammatory pathways, significant gaps remain in translating these findings into clinical practice. In particular, the lack of clinical studies on the optimal dosage, bioavailability, and long-term safety of these dietary components hinders the development of evidence-based nutritional protocols. Consequently, while the use of these dietary components to support tissue regeneration is a promising area, more comprehensive and randomized controlled human studies are needed to clarify dose–response relationships and determine individual nutritional requirements.

### 6.4. Microbiota-Mediated Regulation of Mesenchymal Stem Cell Functions

Mesenchymal stem cells (MSCs) are a promising treatment in regenerative medicine, modulated by age, health status, tissue origin, and many other factors. Recently, it has been revealed that, in addition to host factors, the microbiota living in the human body is also a modulator of MSCs [211]. The microbiota supports epithelial barrier regeneration by regulating the proliferation and differentiation characteristics of intestinal stem cells through microbiota-derived metabolites or microbiota-induced molecules, or via other intestinal cells and immune cells [332,333]. MSCs have been shown to increase blood capillary distribution and tight junction protein expression in the gut, promoting vascularization and barrier function [334]. Furthermore, it has been observed that MSCs restore gut microbiota diversity and function, demonstrating their versatility in regulating immune responses and improving microbiota composition [335]. The interaction between MSCs and the gut microbiota is noteworthy because the gut microbiota plays a crucial role in host metabolism, systemic immune function, and homeostasis of distant organs such as the brain, lungs, liver, and cardiovascular system [336].

The microbiota indirectly regulates MSCs through microorganism components, metabolites, and the host immune system. Lipopolysaccharide (LPS), released from the bacterial cell wall, has been studied as a significant pathogenic factor, particularly in various diseases. In a diabetes mellitus model, high serum LPS levels have been shown to increase the severity of insulin and negate the therapeutic effect of MSC treatment [337]. Microbiota-derived metabolites also play important roles at this point. Short-chain fatty acids increased the differentiation of dental pulp-derived MSCs [338], while propionate suppressed adipogenesis [339]. Other metabolites, trimethylamine N-oxide (TMAO) and D-galactose, reduced MSC proliferation and increased aging [340,341].

Studies have suggested that the microbiota can affect the immunomodulatory capacity of MSCs by creating an inflammatory environment [342,343]. In this context, it has been shown that BMSCs obtained from mice with a normal microbiota cause more T-cell apoptosis compared to cells obtained from germ-free mice. As a result, immunomodulation deficiency and increased inflammation were observed in colitis model mice [344]. Another study showed that MSC treatment reduced colitis symptoms and increased survival in a mouse model of colitis. Furthermore, MSC treatment improved gut microbiota dysbiosis by increasing alpha diversity; raising beneficial bacteria such as Bacteroidetes, Firmicutes, and Tenericutes while reducing harmful bacteria such as Proteobacteria. These findings are consistent with other studies showing that stem cells reduce inflammation and disease symptoms by increasing microbial diversity [345]. A study by Wang et al. in a mouse model of DSS-induced colitis demonstrated that MSC treatment improved colitis symptoms. Treatment reduced pro-inflammatory cytokine levels, restored lymphocyte balance, and improved gut microbiota composition. An increase in beneficial bacteria such as *Firmicutes*, *Lactobacillus*, *Blautia*, *Clostridia*, and *Helicobacter* was reported. Furthermore, MSCs were shown to suppress inflammatory pathways and regulate gene expression associated with ferroptosis. Specifically, increased mucin-1 (MUC-1) expression, elevated levels of SLC7A11 and GPX4 (which protect cells from oxidative damage), and decreased levels of ACSL4 (which enhances ferroptosis) were reported [346]. MSCs regulate the immune response in inflammatory bowel disease (IBD) by secreting anti-inflammatory factors such as IL-10, TGF-β, PGE2, and TSG-6, supporting tissue repair and modulating the gut microbiota. Through this effect, they improve intestinal barrier integrity by increasing intestinal epithelial and stem cell proliferation [18]. The gut microbiota is closely linked to host lipid metabolism, and coordinated alterations in the gut microbiome and lipidome have been identified during the transition from isolated steatosis to non-alcoholic steatohepatitis [347,348]. Human MSC treatment has been reported to improve NASH-related lesions and contribute to the improvement of gut microbiota and metabolic disorders [349].

Exosomes, which play a crucial role in intercellular communication, are shown to be effective in regulating gut microbiota composition and function. MSC-derived exosomes (MSC-exos) restored gut microbiota balance in liver injury and colitis models, increasing beneficial bacteria such as Bacteroides and Parabacteroides and decreasing harmful species [350]. MSC-exos also support intestinal barrier integrity and immune homeostasis by increasing the production of short-chain fatty acids, particularly butyrate [351,352]. Furthermore, MSC-derived exosomes have been reported to improve colitis symptoms by reducing gut bacteria and regulating Th17/Treg balance [353]. These regulatory effects suggest that MSC-exosomes, through their mediation of microbiota and metabolites, could represent a potential novel approach in the treatment of chronic bowel diseases [354,355].

Studies show that microbial imbalance can affect stem cell therapies by disrupting metabolic processes. Staphylococcus aureus has been reported to increase the production of inflammatory mediators such as IL-6 and TNF-α in Wharton’s gel-derived MSCs [356]. Therefore, it is thought that targeting inflammation-related pathways can increase the proliferation and osteogenic differentiation capacity of gingival-derived MSCs (GMSCs) [357]. Dysbiosis in the gut microbiota leads to disruption of the intestinal mucus layer. As a result, bacteria and bacterial products cross the intestinal barrier into the systemic circulation, reaching the pancreas and initiating inflammation and β-cell damage, thereby reducing the effectiveness of ASC treatment [211]. Supporting a healthy microbiota, as well as eliminating a disrupted microbiota, has been shown to improve treatment outcomes. In a mouse model of diabetes mellitus, ASC transplantation has been reported to suppress pancreatitis, protect pancreatic β cells, increase insulin secretion, and reduce blood glucose levels. However, these positive effects were shown to reverse with the restoration of the disrupted diabetic gut microbiota; conversely, suppressing intestinal infection with broad-spectrum antibiotics maintained the treatment effects [337].

Given the impact of the microbiota on MSC treatment, clinical interventions aimed at maintaining the balance of the gut microbiota are being developed; these include optimizing antibiotic use, fecal microbiota transplantation (FMT), administration of probiotics and prebiotics, and direct delivery of microbial metabolic products [334]. Clinical studies have shown that MSC implantation in combination with FMT yields better results in the treatment of IBD because the restoration of microbial homeostasis protected host cells from pathogen invasion and enhanced MSC therapy by aiding in anti-inflammation and tissue remodeling [358].

To understand the mechanism of microbiota regulation on MSCs, experimental models have been created using specific microorganisms and microbial metabolites [359]. Probiotics are also important because of their potential to regulate disrupted microbiota and reduce inflammation [360]. The most used probiotic microorganisms are *Lactobacillus, Bifidobacterium,* and *Enterococcus* species. *Lactobacillus rhamnosus* GG has been shown to protect against bone loss by increasing the proliferation and osteogenesis capacity of MSCs, and to support healing against tissue damage by increasing MSC migration [361,362]. *Lactobacillus reuteri* promoted wound healing by increasing proliferation, differentiation, and migration in MSCs [363]. Similarly, extracts of *Bifidobacterium bifidum* and *Enterococcus faecium* have also been shown to increase the proliferation capacity of MSCs under in vitro conditions [364,365].

In conclusion, the gut microbiota shapes the proliferation, differentiation, and immunomodulatory capacity of MSCs through metabolites, immune signaling, and the inflammatory microenvironment. This interaction suggests that microbiota-targeted interventions (probiotics, FMTs, etc.) could be an important therapeutic strategy in enhancing the effectiveness of MSC-based regenerative therapies.

## 7. Lipidomics and Multi-Omics Integration in Mesenchymal Stem Cell Research

### 7.1. Lipidomic Signatures Specific to Mesenchymal Stem Cells

MSCs are adult stromal cells with multipotent differentiation capacity; they can differentiate into bone, cartilage, and adipose cells and have immunomodulatory properties. MSCs are found in bone marrow, adipose tissue, and umbilical cord [366]. The number of studies showing that cellular lipid composition in stem cells can affect metabolism, behaviors, and fate decisions, thereby causing changes in self-renewal and differentiation capacities, is increasing day by day [367,368,369,370]. Lipids are not only structural components of cellular membranes; they are also molecules that play active roles in signaling, membrane organization, and metabolic regulation processes. The lipidomic signatures of MSCs have a dynamic and complex structure: this lipidomic signature, which includes glycerophospholipids, sphingolipids, and triacylglycerides, may vary depending on the tissue in which MSCs reside, the age of the individual, and environmental stimulants. Membrane lipidomic has a significant effect particularly on stem cell physiology, regulation of stemness, differentiation, and secretion profile [34,122,137,371].

Advances in mass spectrometry-based lipidomic approaches, including untargeted LC–MS techniques, have made it possible to profile lipid species in MSCs and comparative cell types at high resolution. These studies support that MSCs have a cell-specific lipidomic profile by showing that lipidomic signatures differ both between MSCs and other stromal or immune cells such as fibroblasts and peripheral blood mononuclear cells [371,372,373]. Especially, the enrichment of certain phospholipid subclasses in MSCs indicates the presence of a stem cell-specific lipid “fingerprint” [372]. Comparative metabolomic profiling of adipose-derived MSCs, dental pulp-derived MSCs, and human dermal fibroblasts revealed distinct cell-type-related metabolic profiles, supporting metabolomic and lipid profiling as promising approaches for MSC characterization [374]. Phosphatidylglycerol (PG) species are precursors of cardiolipin found in mitochondria, and especially the increase of PG 40:7 in the plasma membrane of MSCs serves as a distinct metabolic marker, making it possible to distinguish MSCs from fibroblasts [375,376,377,378]. Not only lipid composition but also the fatty acid composition within these lipids contributes to MSC identity. A high unsaturated-to-saturated fatty acid (UFA/SFA) ratio is functionally important for stemness. The content of polyunsaturated fatty acids (PUFAs) is important for maintaining membrane fluidity; thus, MSCs can rapidly alter surface receptors and cytoskeletal structures [379,380,381]. With this feature, MSCs can migrate through endothelial gaps to reach injured areas, which is the basis of their homing capacity [381]. Their ability to perform extracellular vesicle (EV) budding from the cell surface and exert paracrine effects is also dependent on this membrane fluidity [137,382,383]. If the cell membrane becomes enriched in SFA, then the membrane begins to stiffen and acts as a physical brake, disrupting these mentioned functions of MSCs. Another important example is the maintenance of the balance between ceramides and sphingosine-1-phosphate (S1P) in MSC lipidome, which is called the sphingolipid rheostat [384,385]. Ceramides show pro-apoptotic/pro-senescence activity, whereas S1P shows pro-survival/pro-proliferative activity, therefore they function like a biological switch [372,386,387,388].

The lipidome of MSCs dynamically changes according to the functional state of the cell. When MSCs transition from a quiescent state to a primed, that is activated state, a metabolic change also occurs in their lipidome, and they begin to exhibit immunomodulatory function [383,389,390]. It has been reported that this change occurs within hours following stimulation with cytokines such as IFN-γ or TNF-α. The shaping of the lipidome proceeds in three ways [391]: (1) membrane lipid content shifts from stable, long-chain saturated lipids to PUFA and PE-O, increasing membrane fluidity and accelerating the release of anti-inflammatory cytokines [380,392,393]; (2) ceramides are rapidly converted into S1P, thereby reshaping the sphingolipid rheostat; this dynamic flux increases the proliferation and migration capacity of the cell and regulates the homing process [394,395,396]; (3) in activated MSCs, membrane PC and phosphatidylserine (PS) are mobilized, thus EV release increases [34,383,397].

Recent studies have confirmed that MSCs possess their own specific lipidomic signatures. This signature plays a role in determining the structural, metabolic, and signaling identity of MSCs. These lipid profiles are dynamic profiles shaped by intrinsic factors such as tissue origin and cellular age, and extrinsic factors such as culture conditions and inflammatory stimulants [34,95,390,398]. Therefore, lipidomic signatures of MSCs should be considered not only as molecular identifiers but also as dynamic indicators reflecting the metabolic and signaling networks of MSCs.

### 7.2. Lipidomic Remodeling During Differentiation

MSCs enter a process of metabolic reprogramming during differentiation, and significant changes are observed especially in their lipidomic profiles [137]. Distinct lipidomic signatures are among the factors that determine which lineage MSCs will commit to [26,158,399,400]. Lipids participate in cellular functions as functional mediators [401]. Changes in the lipid profile are not a consequence of the differentiation process; rather, they are an important remodeling process that plays a role in cell fate decisions by affecting membrane properties, energy utilization, and intracellular signaling [158,402,403]. Lipid profiles undergo dynamic, cell-lineage-specific changes during pluripotent differentiation, with distinct lipid species and classes characterizing neural and mesodermal differentiation trajectories [404]. The transcriptional programs governing adipogenic and osteogenic commitment (PPARγ, C/EBPα, Runx2/Sp7, and the Wnt/β-catenin checkpoint) are described in Section 2.2; here we focus on the accompanying lipidomic remodeling.

Adipogenic differentiation is the differentiation process in which lipidomic remodeling in MSCs is best characterized. During adipogenic differentiation, intracellular neutral lipid accumulation, especially TAGs and diacylglycerols (DAGs), occurs and cytoplasmic lipid droplets (LD) are formed [70,405]. During differentiation, the amount of neutral lipids, especially in triglyceride (TG) form, which is around 10% in MSCs, reaches up to 97% during the process [406]. While TGs accumulate within the cell, phospholipid composition also changes; PCs, PEs, and lysophospholipids (LPC/LPE) increase, supporting the formation of the phospholipid monolayer surrounding LDs [406]. DAGs act as precursors in TG synthesis; their levels increase especially in the early–mid stages of differentiation and contribute to LD formation [405,406]. LDs are not only lipid storage units but are also dynamic and function as metabolic hubs; they store enzymes such as lipases and play roles in the regulation of signaling pathways related to cell proliferation and differentiation [405,407,408].

In contrast, during the differentiation of MSCs toward the osteogenic lineage, lipid utilization rather than storage is at the forefront [409]. Osteogenesis requires the breakdown of intracellular lipids, and the provision of extra energy needed for matrix synthesis and mineralization processes; remodeling of the lipid profile in differentiating MSCs occurs in this context [67,409,410]. Accordingly, intracellular LD levels decrease, and mitochondrial fatty acid oxidation (FAO) increases during the maturation of osteoprogenitors to meet the high ATP demand of the cell [67,410,411]. During osteogenesis, catabolic activities in MSCs increase toward the breakdown of TG content, and anabolic pathways such as Wnt/ß-catenin promote FAO and suppress lipid accumulation [410,411,412]. These studies underline the importance of metabolic switches in MSC fate decisions [413].

Another cell group into which MSCs differentiate is chondrocytes; it has been reported that lipid composition in MSCs also changes during chondrogenesis, thereby regulating cell structure, function, and signaling pathways [414]. In the early stages of chondrogenesis, the levels of sphingolipids such as phosphocholine, sphingomyelins (SM), and PCs increase [414].

The key differences in lipid remodeling, metabolic strategies, and transcriptional regulators between adipogenic and osteogenic differentiation are summarized in Table 3.

Lipidomic remodeling also affects the cell at the mechanical level, creating a response in the cell like a physical stimulus by playing a role in changing the biophysical properties of the cell membrane. This process is shaped by the reprogramming of different lipid classes in the membrane [404,415,416,417]. Membrane fluidity and stiffness are determined by lipid content; during differentiation, an increase in saturated (SFA) and monounsaturated (MUFA) fatty acids in membrane lipids affects membrane fluidity [401,416,418,419]. Microdomains on the membrane called lipid rafts are remodeled by the distribution of cholesterol contained in the membrane and by products resulting from lipid oxidation [415,417]. Changes in the lipid profile of the cell also regulate the functions of transcription factors; for example, the expression of SREBPs is regulated by phospholipid composition on the ER (endoplasmic reticulum) membrane and dictates lipid synthesis during differentiation [420]. Lipid intermediates such as ceramide, DAG, and lysophosphatidylcholine (LPC) function as secondary messengers in signaling pathways and take roles in signaling pathways active during differentiation [421]. PUFA and eicosanoid metabolites are lipid-derived ligands and function in PPAR activation; these are the ligands that enable transcription factors governing adipogenic differentiation to function [422]. While PPARγ acts as a master regulator in adipogenesis, PPARα regulates FAO and ketosis under energy deprivation conditions [422]. Other lipid mediators, including phospholipids and sphingolipids, modulate Wnt, TGF-β, and mTOR signaling [423,424,425], integrating lipid remodeling with pathways that coordinate differentiation and metabolism (Section 2.2 and Section 4.3).

### 7.3. Multi-Omics Integration of Diet–Lipid–MSC Interaction

Multi-omics approaches have made it possible to understand and evaluate the roles of lipids in metabolic, inflammatory, and differentiation-related pathways from a systems perspective [426,427]. Transcriptomic studies examining changes at the gene level and epigenomic analyses examining diet-dependent changes in epigenetic patterns such as DNA methylation and histone modifications contribute to understanding how diet and lifestyle affect metabolic phenotypes in the long term [428,429,430,431]. Proteomic analyses, used to examine changes at the functional level, are used especially to determine changes in metabolic enzyme levels and to examine the levels of proteins involved in intracellular signaling processes [432]. Lipidomic analyses, one of the most used omics approaches in diet-based studies, enable the direct measurement and identification of lipid content. The holistic evaluation of all these omics analyses together has enabled researchers to comprehensively assess the cellular effects of diet [426,427,431,432,433,434].

Multi-omics approaches such as transcriptomics, proteomics, and lipidomics have recently begun to be widely used in studies to understand how metabolic changes in MSCs are translated into functional outputs [435,436,437,438]. Lipidomic studies have been extremely useful in understanding how dietary changes affect cellular behaviors and how they affect the differentiation dynamics of MSCs [438]. The dietary content and nutritional status of individuals are upstream regulators of cellular lipid composition. As explained in previous sections, the lipid profile of the diet affects the lipid profile and function of MSCs [34]. Diets rich in saturated fatty acids cause an increase in biologically active lipid species such as ceramides in the cell and are associated with lipotoxicity, inflammation, and cellular dysfunction. Especially such diets affect MSC reserves in the bone marrow, favor adipogenic commitment, and increase the risk of obesity development in the individual [67,439,440,441]. In individuals fed with diets rich in omega-3 PUFAs, it has been reported that anti-inflammatory lipid mediators increase. Mediators such as resolvins and maresins contribute to the remodeling of the membrane lipid structure of MSCs, increase their proliferative capacity, and promote osteogenic differentiation [158,168].

Advanced computational tools are extremely crucial for the comprehensive evaluation of the data obtained from the multi-omics analyses. In particular, the evaluation of independent omics datasets with these advanced tools is used to identify new molecular biomarkers and signatures [442,443,444].

Advanced computational tools have further strengthened this integrative approach. Methods such as Multi-Omics Factor Analysis (MOFA), DIABLO (Data Integration Analysis for Biomarker discovery using Latent cOmponents), and network-based modeling enable the identification of shared patterns and causal relationships across different omics layers. These approaches are particularly valuable in distinguishing correlation from causality and allow the identification of critical regulatory nodes that drive MSC function. In this context, lipid metabolism often emerges as a central node linking dietary inputs to transcriptional and functional outputs. MOFA extends principal component analysis (PCA) of multi-omics data and has a data-driven structure. It performs analysis and integration of data obtained from different omics layers such as somatic mutations, DNA methylation, and RNA expression both jointly and within their own layers [445]. DIABLO identifies different phenotypic groups by extracting correlations between different datasets such as mRNA, miRNA, and proteins [444]. Network-based approaches include methods such as Similarity Network Fusion (SNF) or graph-based approaches that map molecular interactions, thereby enabling the emergence of new network topologies by integrating data into predefined biological pathways [446].

The diet–lipid–MSC interaction is mediated by regulatory nodes such as lipid trafficking pathways, inter-organelle interactions, and metabolic compartmentalization [95,447]. In particular, the spatial organization of lipids within the cell is extremely critical in cellular architecture and affects the self-renewal and differentiation capacities of stem cells [448]. Crosstalk between intracellular organelles and membrane–membrane contact sites is regulated through the transfer of lipids and signaling molecules [449,450,451]. In this framework, it can be said that dietary lipids directly play a role in both signaling and intracellular compartmentalization of MSCs. The use of multi-omics approaches contributes to elucidating the diet–lipid–MSC axis and to understanding the effect of lipid metabolism on stem cells [452,453].

## 8. Conclusions and Future Directions

In conclusion, recent advances in mass spectrometry-based lipidomics have facilitated the characterization of MSC-specific lipidomic signatures, providing valuable insights into the metabolic mechanisms governing stemness, differentiation, and regenerative function. Lipids function not only as energy sources and structural components of cellular membranes. The biological functions of MSCs, including proliferation, differentiation, immunomodulation, and tissue regeneration, are closely governed by metabolic processes such as fatty acid uptake, β-oxidation, de novo lipid synthesis, and membrane remodeling. In MSCs, fatty acid uptake is mediated by specific transport proteins, including CD36. Elevated expression of CD36 facilitates fatty acid uptake and cytosolic transport, thereby contributing to metabolic reprogramming that influences cell fate decisions and favors adipogenic differentiation. Another important factor influencing stem cell fate is the balance between lipogenesis and lipid oxidation. Whereas fatty acid oxidation regulates energy homeostasis and metabolic reprogramming, de novo lipogenesis supplies lipids required for membrane synthesis during proliferation and differentiation. Together, these processes contribute to the maintenance of stemness and the regulation of lineage-specific differentiation.

In addition to these intracellular metabolic pathways, lipid-derived signaling mediators, such as eicosanoids particularly PGE2, further extend the regulatory role of lipids by modulating MSC immunomodulatory activity, migratory capacity, and tissue repair functions in a microenvironment-dependent manner. MSCs also express enzymes required for the biosynthesis of SPMs, indicating that these lipid mediators contribute to their anti-inflammatory and immunoregulatory effects. Moreover, lipid-derived signaling mediators regulate MSC fate by activating nuclear receptors such as PPARγ, PPAR-β/δ, LXRα/β, and RXR. Activation of these receptors modulates gene expression programs that drive lineage commitment, promoting adipogenic and osteogenic differentiation while influencing overall MSC functional plasticity. Beyond their roles in regulating proliferation and differentiation, lipid metabolism and lipid-derived signaling mediators are also involved in key cellular processes underlying MSC function. For example, activation of the PPAR-δ signaling pathway increases the oxidation of free fatty acids, which may promote asymmetric division of MSCs and thereby contributes to the preservation of the MSC pool. Lipid rafts are dynamic, cholesterol- and sphingolipid-rich membrane microdomains that cluster in response to stimuli to form signaling platforms, thereby facilitating cell–environment interactions and regulating key signaling pathways involved in stem cell function. Moreover, several lipid metabolism-related genes have been identified as key regulators of MSC behavior and epigenetic modifications. However, further mechanistic studies are needed to elucidate how specific lipid species and lipid-derived mediators dynamically regulate nuclear receptor signaling and epigenetic remodeling in MSC fate decisions.

Diet and dietary factors-driven lipid-mediated metabolic pathways represent a crucial factor that can also modulate MSC function. Therefore, use of multi-omics approaches contributes to elucidating the diet–lipid–MSC axis and to understanding the effect of lipid metabolism on stem cells. Caloric restriction has been shown to protect MSCs through metabolic reprogramming, preservation of regenerative function, and attenuation of age-related cellular deterioration. Micronutrients and phytochemicals support MSC homeostasis by promoting a microenvironment conducive to MSC survival, proliferation, and differentiation. Furthermore, gut microbiota shapes the proliferation, differentiation, and immunomodulatory capacity of MSCs through metabolites, immune signaling, and the inflammatory microenvironment. This interaction suggests that microbiota-targeted interventions could be an important therapeutic strategy in enhancing the effectiveness of MSC-based regenerative therapies. In contrast, obesity and high-fat diets have been associated with reduced regenerative capacity, enhanced adipogenic differentiation, and impaired cellular homeostasis in MSCs.

Based on the current evidence, we propose an integrative diet–lipidome–MSC axis in which dietary factors and gut microbiota influence lipid metabolism and lipid-derived signaling, which in turn regulate MSC stemness, differentiation, immunomodulation, and regenerative functions. This proposed model highlights lipid metabolism as a potential link between nutritional status and MSC function. However, current evidence remains limited. Future studies should focus on standardized lipidomic profiling of MSCs, identification of source-specific lipid signatures, and assessment of donor metabolic status. Optimization of lipid composition in MSC culture media, as well as dietary and microbiota-based MSC conditioning, should also be investigated. Single-cell lipidomic profiling of patient-derived MSCs and personalized nutritional interventions may provide further insights into individual differences in MSC metabolism. Lipid-targeted small-molecule adjuvants could also be explored to improve MSC-based therapies. Finally, randomized human studies are needed to better understand nutrition–MSC interactions and their potential role in regenerative medicine.

## Figures and Tables

**Table 1:** Experimental findings of lipid-metabolic pathways and their associated outcomes in MSC models.

Lipid Pathway	Model/Cell Type	Key Findings	Main Finding	Refs.
**Fatty Acid** **Uptake**	Hypoxic pancreatic β-cells & BM-MSCs co-culture	↑ Beta cell viability ↑ Cell survival	BMSC/EV-mediated CD36 gene downregulation → ↓ Lipid uptake, oxidative stress, and metabolic dysfunction	[100]
	ApoE-knockout mouse model & macrophages	↓ Foam cell formation	BM-MSC-driven CD36 & SRA downregulation → Blockade of oxLDL uptake → ↓ intracellular lipid accumulation	[101]
**Fatty Acid** **Oxidation**	MSC administration in renal ischemia/reperfusion injury rat models	Attenuation of acute kidney injury post-reperfusion	Metabolic shift towards lipid catabolism → ↓ Lipid peroxidation products (MDA and 4-HNE)	[112]
	NASH mouse models & hUC-MSC & HepG2 co-culture	↓ Intracellular lipid and TAG accumulation in hepatocytes	MSC-hepatocyte tunneling nanotube (TNT) formation → Healthy mitochondrial transfer → ↑ Cellular β-oxidation	[113,114]
	90% hepatectomy liver failure model & PL-MSC experimental lines	↓ Hepatic steatosis & lipid accumulation	mTOR cascade stimulation → upregulation of CPT1A rate-limiting expression → ↑ Cellular β-oxidation	[115,116]
**De Novo** **Lipogenesis**	C57BL/6 male mouse liver model & BM-MSCs administration	↓ Hepatic lipid accumulation	↓ Hepatic SCD1, and SREBP-2 expression → Suppressed de novo hepatic lipogenesis pathway	[117]
	Murine AT-MSC & macrophage co-culture model	Induction of anti-inflammatory M2 phenotype	AT-MSC conditioned medium (CM) activates macrophage AKT/mTOR phosphorylation → Drives both lipid droplet biogenesis	[120]

↑, increase; ↓, decrease. AMPK, AMP-activated protein kinase; AT-MSCs, adipose tissue-derived mesenchymal stem cells; BM-MSCs, bone marrow-derived mesenchymal stem cells; BMSCs, bone marrow stem cells; CD36, cluster of differentiation 36; CPT, carnitine palmitoyltransferase; CSCs, cancer stem cells; DNL, de novo lipogenesis; EVs, extracellular vesicles; FAS/FASN, fatty acid synthase; HER2, human epidermal growth factor receptor 2; HIF-1α, hypoxia-inducible factor-1 alpha; IDO, indoleamine 2,3-dioxygenase; LPL, lipoprotein lipase; M1/M2, macrophage phenotypes; MSCs, mesenchymal stem cells; mTOR, mechanistic target of rapamycin; NASH, non-alcoholic steatohepatitis; oxLDL, oxidized low-density lipoprotein; SCD1, stearoyl-CoA desaturase-1; SRA, class A scavenger receptor; SREBP1/2, sterol regulatory element-binding protein 1/2; STAT, signal transducer and activator of transcription; TAGs, triacylglycerols (triglycerides); TNF-α, tumor necrosis factor-alpha; UC-MSCs, umbilical cord-derived mesenchymal stem cells; Wnt, wingless-related integration site. Note: Rows are categorized by the primary lipid pathways discussed in the text, detailing specific experimental models, cell lines, and corresponding outcomes.

**Table 2:** Key modulators of mesenchymal stem cell function.

Category	Reported Effects on MSCs	Mechanism/Pathway	Refs.
*VITAMINS*			
Vitamin A	↑ immunosuppression; ↓ T-cell activity	Regulation of SIRT1, Wnt, NF-κB, and Nrf2 signaling	[298,299]
Vitamin B_3_	↑ MSCs survival; ↓ replicative senescence; ↑ differentiation	NAD^+^-dependent metabolic pathways and regulation of cellular senescence	[310]
Vitamin B_6_	↑ immunomodulatory potential; ↑ clearance of MSCs from the body	One-carbon metabolism and epigenetic regulation through DNA and histone methylation	[308,309]
Vitamin C	↑ ROS scavenging; ↑ proliferation & self-renewal; ↑ osteogenic/chondrogenic differentiation	Antioxidant activity and regulation of proliferation- and differentiation-related gene expression	[302,303]
Vitamin D	↓ senescence; ↑ osteogenic differentiation; ↓ inflammation	Vitamin D-mediated regulation of stemness, senescence, proliferation, and inflammatory pathways	[304,305,306]
Vitamin E	↓ oxidative stress; ↓ apoptosis; ↑ cartilage regeneration	Antioxidant activity and modulation of cell-survival signaling	[303,307]
Vitamin K_2_	↑ osteogenic potential (synergistic with vitamin D)	Regulation of osteogenic pathways and interaction with vitamin D signaling	
*MINERALS*			
Calcium	↑ proliferation; ↑ osteogenic differentiation	Ca^2+^-dependent signaling and osteo-inductive activity; regulation of Wnt/β-catenin signaling	[318,319,321]
Magnesium	↑ proliferation; ↑ adhesion; ↓ inflammation ↑ osteo-/chondrogenic potential;	Regulation of cellular signaling, proliferation, adhesion, and differentiation	[311,312,313,314,315]
Selenium	↑ cell survival; ↓ senescence; ↑ osteogenic potential; ↓ inhibit adipogenesis; ↓ oxidative stress	Regulation of redox homeostasis and antioxidant defense	[313,317]
Zinc	↑ proliferation; ↑ osteogenesis; ↑ antioxidant defense; ↑ tissue repair	Regulation of proliferation, telomerase-related gene expression, and antioxidant responses	[316]
*PHYTOCHEMICALS*			
EGCG	↓ oxidative stress; ↓senescence (anti-aging); ↑ osteogenic differentiation; ↓ inflammation	Antioxidant, anti-inflammatory, and osteogenic signaling pathways	[327,328,329]
Curcumin	↓ oxidative stress; ↓ apoptosis; ↓ inflammation	Modulation of oxidative stress, apoptotic, and inflammatory signaling pathways	
Allicin	↑ osteogenesis; ↑ bone remodeling	Regulation of osteogenic signaling and bone-remodeling processes	
Resveratrol	↑ proliferation; ↑ osteogenic differentiation	Modulation of intracellular signaling pathways involved in proliferation and osteogenic differentiation	
Coenzyme Q10	↓ senescence; ↓ oxidative stress; ↓ aging markers	Mitochondrial antioxidant activity and regulation of mitochondrial quality control	
Melatonin	↑ survival; ↑ proliferation; ↓ inflammation ↓ intracellular stress & damage; ↓ apoptosis;	Regulation of oxidative stress, apoptosis, mitochondrial function, and cell-survival pathways	

Abbreviation MSC: mesenchymal stem cell; ROS: reactive oxygen species. ↑ indicates an increase or upregulation, whereas ↓ indicates a decrease or downregulation in the reported biological effect or cellular process.

**Table 3:** Key lipidomic and metabolic differences between adipogenic and osteogenic differentiation of MSCs.

Feature	Adipogenic Differentiation	Osteogenic Differentiation
Net lipid strategy	Storage—accumulation of neutral lipids (TAG, DAG) and formation of cytoplasmic LDs	Utilization—breakdown of intracellular lipids to fuel matrix synthesis and mineralization
Neutral lipid content	Triglycerides rise from ~10% to up to 97% of cellular lipid during differentiation	Intracellular lipid-droplet levels decrease as differentiation proceeds
Master transcription factors	PPARγ (master regulator) and C/EBPα; SREBP1 drives de novo lipogenesis and generates endogenous PPARγ ligands, forming a positive-feedback loop	Runx2 and Sp7 (osterix); Wnt/β-catenin promotes FAO and suppresses lipid accumulation
Energy metabolism	Lipid storage predominates; LDs act as dynamic metabolic hubs that store lipases and modulate proliferation/differentiation signaling	Mitochondrial FAO increases to meet the high ATP demand of maturing osteoprogenitors
Phospholipid remodeling	↑ PC, PE and lysophospholipids (LPC/LPE), supporting the phospholipid monolayer around lipid droplets	Membrane remodeling with increased SFA and MUFA fatty acids affecting membrane fluidity

↑ increased/upregulated. TAG, triacylglycerol; DAG, diacylglycerol; LD, lipid droplet; PPARγ, peroxisome proliferator-activated receptor gamma; C/EBPα, CCAAT/enhancer-binding protein alpha; SREBP1, sterol regulatory element-binding protein 1; Runx2, runt-related transcription factor 2; Sp7, osterix; FAO, fatty acid oxidation; ATP, adenosine triphosphate; PC, phosphatidylcholine; PE, phosphatidylethanolamine; LPC, lysophosphatidylcholine; LPE, lysophosphatidylethano-lamine; SFA, saturated fatty acid; MUFA, monounsaturated fatty acid; Wnt, wingless-related integration site.

## Data Availability

No new data were created or analyzed in this study. Data sharing is not applicable to this article.

## Abbreviations

The following abbreviations are used in this manuscript:

1-MGs	1-monoglycerides
AAs	amino acids
Akt/PKB	protein kinase B
ALA	alpha-linolenic acid
ALI	acute lung injury
ALP	alkaline phosphatase
AM-MSCs	amniotic membrane-derived MSCs
AMPK	AMP-activated protein kinase
Ang	angiopoietin
ANGPTL4	angiopoietin-like 4
ARA	arachidonic acid
AT-MSCs	adipose tissue-derived MSCs
ATGL	adipose triglyceride lipase
ATP	adenosine triphosphate
BM-MSCs	bone marrow-derived MSCs
BMP	bone morphogenetic protein
CACT	carnitine-acylcarnitine translocase
cAMP	cyclic adenosine monophosphate
CCL2	chemokine (C-C motif) ligand 2
CD36	cluster of differentiation 36
CD117	cluster of differentiation 117
C/EBP	CCAAT/enhancer-binding proteins (α, β, δ)
CFU-F (CFC-F)	colony-forming unit–fibroblast
COX-2	cyclooxygenase-2
CPT	carnitine palmitoyltransferase
CR	caloric restriction
CREB	cyclic AMP response element-binding protein
CXCL1	C-X-C motif chemokine ligand 1
CXCR4	C-X-C chemokine receptor type 4
DAGs	diacylglycerols
Dgat2	diacylglycerol O-acyltransferase 2
DHA	docosahexaenoic acid
DNL	de novo lipogenesis
DP-MSCs (DPSCs)	dental pulp-derived MSCs
ECM	extracellular matrix
EGF	epidermal growth factor
ELOVL6	elongation of very long chain fatty acids protein 6
EP2	prostaglandin E2 receptor 2
EPA	eicosapentaenoic acid
ERK	extracellular signal-regulated kinase
ES	embryonic stem (cells)
EV	extracellular vesicle
FABP4/aP2	fatty acid-binding protein 4/adipocyte protein 2
FABPpm (GOT2)	plasma membrane fatty acid-binding protein/ glutamic-oxaloacetic transaminase 2
Fads2	fatty acid desaturase 2
FAO	fatty acid oxidation
FAs	fatty acids
FAS/FASN	fatty acid synthase
FFAR4	free fatty acid receptor 4
FFAs	free fatty acids
FGFs	fibroblast growth factors
FMT	fecal microbiota transplantation
FOXO	forkhead box O
GLA	gamma-linolenic acid
GLUT4	glucose transporter type 4
GMSCs	gingival-derived MSCs
GPCRs	G protein-coupled receptors
GPIHBP1	glycosylphosphatidylinositol-anchored HDL-binding protein 1
GPL	glycerophospholipid
GRs	glucocorticoid receptors
hAMSCs	human amniotic MSCs
HER2	human epidermal growth factor receptor 2
HFD(s)	high-fat diet(s)
Hh	Hedgehog
HGF	hepatocyte growth factor
HIF-1α	hypoxia-inducible factor-1α
HIFs	hypoxia-inducible factors
HLA-DR	human leukocyte antigen-DR
HLA-G5	human leukocyte antigen-G5
hMSCs	human MSCs
HSPC	hematopoietic stem and progenitor cell
IBD	inflammatory bowel disease
ICAM-1	intercellular adhesion molecule-1
IDO	indoleamine 2,3-dioxygenase
IFN-γ	interferon-γ
IGF/IGF-1	insulin-like growth factor/-1
IL	interleukin
IL-1Ra	IL-1 receptor antagonist
ISCs	intestinal stem cells
ISCT	International Society for Cellular Therapy
JAK	Janus kinase
JNK	c-Jun N-terminal kinase
Klf4/KLFs	Krüppel-like factor 4/factors
LA	linoleic acid
LD	lipid droplets
LDH	lactate dehydrogenase
LDL/oxLDL	low-density lipoprotein/oxidized LDL
LPC	lysophosphatidylcholine
LPL	lipoprotein lipase
LPS	lipopolysaccharide
LTB4	leukotriene B4
LXR	liver X receptor
M-CSF	macrophage colony-stimulating factor
MCP-1	monocyte chemoattractant protein-1
MHCII	major histocompatibility complex class II
miRNAs	microRNAs
MOFA	Multi-Omics Factor Analysis
MRP4	multidrug resistance protein 4
MSC(s)	mesenchymal stem/stromal cell(s)
MSC-exos	MSC-derived exosomes
MT1-MMP	membrane-type 1 matrix metalloproteinase
mTOR	mechanistic target of rapamycin
MUC-1	mucin-1
MUFA(s)	monounsaturated fatty acids
NAD^+^	nicotinamide adenine dinucleotide
NASH	non-alcoholic steatohepatitis
NK	natural killer (cells)
NO	nitric oxide
PC	phosphatidylcholine
PCA	principal component analysis
PD-L1	programmed death-ligand 1
PL-MSCs	placenta-derived MSCs
PDGF	platelet-derived growth factor
PE	phosphatidylethanolamine
PE-O	ether-linked phosphatidylethanolamines
Pfn-1	profilin-1
PG	phosphatidylglycerol
PGE2	prostaglandin E2
PI	phosphatidylinositol
PI3K	phosphoinositide 3-kinase
PI3K/AKT/mTOR	phosphoinositide 3-kinase/protein kinase B/mechanistic target of rapamycin
PLA2	phospholipase A2
PLC-β2	phospholipase C beta 2
PPARα	peroxisome proliferator-activated receptor alpha
PPARγ	peroxisome proliferator-activated receptor gamma
PPAR-β/δ	peroxisome proliferator-activated receptor beta/delta
PS	phosphatidylserine
PTH	parathyroid hormone
PUFAs	polyunsaturated fatty acids
RANKL	receptor activator of nuclear factor kappa-B ligand
ROS	reactive oxygen species
Runx2	runt-related transcription factor 2
RXR	retinoid X receptor
S1P	sphingosine-1-phosphate
SCAP	SREBP cleavage-activating protein
SCD1/SCD2	stearoyl-CoA desaturase-1/-2
SCF	stem cell factor
scWAT	subcutaneous white adipose tissue
SDF-1	stromal cell-derived factor-1
SFA(s)	saturated fatty acids
SIRT1	sirtuin 1
SM	sphingomyelin
SM-MSCs	synovial membrane-derived MSCs
SNF	Similarity Network Fusion
Sox9	SRY-box containing gene 9
Sp7	osterix
SPMs	specialized pro-resolving mediators
SRA	class A scavenger receptor
SREBF1c/SREBP	sterol regulatory element-binding factor-1c/protein
STAT	signal transducer and activator of transcription
TAGs	triglycerides
TAK1	TGF-β-activated kinase 1
TCR	T-cell receptor
TGF-β	transforming growth factor-beta
TMAO	trimethylamine N-oxide
TNF-α	tumor necrosis factor-α
TNTs	tunneling nanotubes
TSG-6	TNF-stimulated gene-6
UC-MSCs	umbilical cord-derived MSCs
UCB	umbilical cord blood
UFA/SFA	unsaturated-to-saturated fatty acid (ratio)
VCAM-1	vascular cell adhesion molecule-1
VEGF	vascular endothelial growth factor
VLA-4	very late antigen-4
VLDL	very low-density lipoproteins
VAT	visceral adipose tissue
WAT	white adipose tissue
Wnt	Wingless-type MMTV integration site
